# Theranostic Design of Angiopep-2 Conjugated Hyaluronic Acid Nanoparticles (Thera-ANG-cHANPs) for Dual Targeting and Boosted Imaging of Glioma Cells

**DOI:** 10.3390/cancers13030503

**Published:** 2021-01-28

**Authors:** Angela Costagliola di Polidoro, Giorgia Zambito, Joost Haeck, Laura Mezzanotte, Martine Lamfers, Paolo Antonio Netti, Enza Torino

**Affiliations:** 1 Department of Chemical, Materials and Production Engineering (DICMaPI), University of Naples Federico II, 80125 Naples, Italy; angela.costaglioladipolidoro@unina.it (A.C.d.P.); nettipa@unina.it (P.A.N.); 2 Fondazione Istituto Italiano di Tecnologia, IIT, 80125 Naples, Italy; 3 Department of Molecular Genetics, Erasmus Medical Center, 3015 CN Rotterdam, The Netherlands; g.zambito@erasmusmc.nl (G.Z.); l.mezzanotte@erasmusmc.nl (L.M.); 4 Medres Medical Research GmBH, 50931 Cologne, Germany; 5 Department of Radiology and Nuclear Medicine, Erasmus Medical Center, 3015 CN Rotterdam, The Netherlands; 6 AMIE Core Facility, Erasmus Medical Center, 3015 CN Rotterdam, The Netherlands; j.haeck@erasmusmc.nl; 7 Department of Neurosurgery, Brain Tumor Center, Erasmus Medical Center, 3015 CN Rotterdam, The Netherlands; m.lamfers@erasmusmc.nl; 8 Interdisciplinary Research Center on Biomaterials, CRIB, University of Naples Federico II, 80125 Naples, Italy

**Keywords:** glioblastoma, theranostics, angiopep-2, BBB, active targeting, irinotecan, precision medicine, MRI, hyaluronic acid, hydrodenticity, nanomedicine

## Abstract

**Simple Summary:**

Glioblastoma multiforme is the most aggressive malignant brain tumor with poor patient prognosis. The presence of the blood-brain barrier and the complex tumor microenvironment impair the efficient accumulation of drugs and contrast agents, causing late diagnosis, inefficient treatment and monitoring. Functionalized theranostic nanoparticles are a valuable tool to modulate biodistribution of active agents, promoting their active delivery and selective accumulation for an earlier diagnosis and effective treatment, and provide simultaneous therapy and imaging for improved evaluation of treatment efficacy. In this work, we developed angiopep-2 functionalized crosslinked hyaluronic acid nanoparticles encapsulating gadolinium-diethylenetriamine pentaacetic acid (Gd-DTPA) and irinotecan (Thera-ANG-cHANPs) that were shown to boost relaxometric properties of Gd-DTPA by the effect of Hydrodenticity, improve the uptake of nanoparticles by the exploitation of angiopep-2 improved transport properties, and accelerate the therapeutic effect of Irinotecan.

**Abstract:**

Glioblastoma multiforme (GBM) has a mean survival of only 15 months. Tumour heterogeneity and blood-brain barrier (BBB) mainly hinder the transport of active agents, leading to late diagnosis, ineffective therapy and inaccurate follow-up. The use of hydrogel nanoparticles, particularly hyaluronic acid as naturally occurring polymer of the extracellular matrix (ECM), has great potential in improving the transport of drug molecules and, furthermore, in facilitatating the early diagnosis by the effect of hydrodenticity enabling the T_1_ boosting of Gadolinium chelates for MRI. Here, crosslinked hyaluronic acid nanoparticles encapsulating gadolinium-diethylenetriamine pentaacetic acid (Gd-DTPA) and the chemotherapeutic agent irinotecan (Thera-cHANPs) are proposed as theranostic nanovectors, with improved MRI capacities. Irinotecan was selected since currently repurposed as an alternative compound to the poorly effective temozolomide (TMZ), generally approved as the gold standard in GBM clinical care. Also, active crossing and targeting are achieved by theranostic cHANPs decorated with angiopep-2 (Thera-ANG-cHANPs), a dual-targeting peptide interacting with low density lipoprotein receptor related protein-1(LRP-1) receptors overexpressed by both endothelial cells of the BBB and glioma cells. Results showed preserving the hydrodenticity effect in the advanced formulation and internalization by the active peptide-mediated uptake of Thera-cHANPs in U87 and GS-102 cells. Moreover, Thera-ANG-cHANPs proved to reduce ironotecan time response, showing a significant cytotoxic effect in 24 h instead of 48 h.

## 1. Introduction

Glioblastoma multiforme (GBM) or grade IV astrocytoma is the most aggressive malignant brain tumor [1]. In 2017 the European Association of Neuro Oncology (EANO) published the latest guidelines for GBM diagnosis, tumour volume evaluation and therapy follow up defining MRI as the gold standard [2] and the radiographic appearance of GBM as the presence of enhancing regions surrounding a dark, necrotic core in T1 post-contrast images [3].

GBM standard of care is defined by the “Stupp Protocol” as postoperative chemotherapy with the alkylating agent temozolomide (TMZ) in combination with radiotherapy [2]. However, TMZ has been proven to be beneficial in less than 50% of patients and, in particular, those affected by the methylation of the O (6)-methylguanine-DNA methyltransferase (MGMT) promoter. Also, GBM cells bear intrinsic and adaptive TMZ resistance mechanisms which strongly reduce its efficacy [4].

In 2006, the United States Food and Drug Administration (FDA) approved the antiangiogenic monoclonal antibody bevacizumab as adjuvant therapy in patients with severe edema [5] without, however, conferring any benefit in patient prognosis and long term survival, which remain both very poor. Indeed, this pathology is characterized by a late diagnosis, absence of a precise evaluation of treatment efficacy and accuracy of the follow-up, and inability to prevent tumour recurrence [5] with a patient mean survival of only 15 months. The presence of the blood-brain barrier (BBB) and the very distinct landscape characterizing GBM microenvironment made of physical [6,7,8], chemical [9], mechanical [10,11] and biological [12,13,14] barriers, prevent the accumulation of contrast agents, drugs at therapeutic concentrations, and decrease their therapeutic potential because of multidrug resistance development [9,13,14]. The enhancing portion of the tumor mass observed by MRI is the result of the diffusion of active molecules into the perinecrotic regions of the tumor mass since they are characterized by newly formed and leaky blood vessels with impaired BBB function [15]. GBM diagnosis, treatment and monitoring may therefore be significantly delayed depending on the vascularization of the tumor and may exclude a significant portion of the total mass. In addition, in almost 30% of patients, chemo-radiotherapy causes pseudoprogression, the radiographic appearance of radiation-related necrosis which is simulating progressive disease, potentially misleading to therapy interruptions or modifications [16,17]. Similarly, antiangiogenic therapy may cause pseudoresponse, a reduction of the enhancing regions because of edema alleviation, without any significant variation in the actual tumor mass [16,17].

For these reasons, there is an urgent need for techniques and tools to give a more precise diagnosis and distinguish between the pseudoprogression of the radiotherapy and the recurrence of the pathology. In this regard, perfusion and diffusion-based sequences, such as T1 weighted Dynamic Contrast-Enhanced (DCE)—MRI, are posing as a promising alternative since they provide an accurate characterization of the tumor microenvironment through the estimation of pathophysiological parameters of the extracellular matrix and in this way, they successfully reveal the presence of residual tumor [18]. However, the estimation of these parameters still suffers from poor reproducibility because of the reduced spatial resolution of images acquired to follow the very rapid diffusion of contrast agent (CA) molecules across highly leaky blood vessels [19].

In this framework, nanoparticle-based formulations can play a role in controlling biodistribution, transport properties and transport mechanisms of the active agents for therapy and diagnosis. Nanoparticles are able to improve drug bioavailability, protect CAs molecules from transmetalation, and, when properly engineered, target active agent molecules specifically at the diseased site using active targeting mechanisms [20]. Indeed, the selection of ad hoc targeting moieties can both trigger specific internalization mechanisms as receptor mediated transcytosis and avoid active agents being substrates of P-efflux pumps, responsible for the major GBM resistance to therapy. In addition, nanoparticles offer a unique possibility to co-deliver simultaneously multiple active agents to enable multimodal imaging and theranostics. These approaches may contribute to effective therapy, earlier diagnosis and more accurate evaluation of treatment efficacy during the follow-up, potentially improving patient outcomes.

Recently, in 2019, Lux et al. initiated a Phase II Clinical trial with the theranostic formulation AguiX for simultaneous Brain Radiotherapy and Imaging (NCT03818386). They applied ultrasmall polysiloxane matrices (<5 nm) decorated with 10 molecules of Gd-DOTAGA as radiosensitizer and CA for brain metastasis [21]. However, the ultrasmall size, which may improve nanoparticle accumulation at the diseased site, may similarly favour a very rapid clearance from the tumour mass, significantly reducing the window both for therapy and image acquisition. Furthermore, the formulation proposes a chemical modification of the Gd chelates, as approved in the clinical practice, that may fall into the destabilization and transmetalation, inducing Gd ion liberation with consequent very high toxicity. Also, polysiloxanes have limited biocompatibility because of their hydrophobic nature that can bring irreversible and undesirable adsorption of nonpolar molecules, bimolecular species, and cells to their surfaces [22]. Finally, several works [6,23,24] are showing the minimal efficacy of the enhanced retention and permeability (EPR) effect in favouring selective accumulation of nanoparticles at the tumor site, above all in the brain. For this reason, a strategy to guarantee sufficient active agent crossing preferably the endothelial layer of the healthy vasculature of the tumor needs to be developed. This evidence becomes even more relevant in GBM where almost 80% of the tumour mass is characterized by a healthy BBB [25], which is further limiting passive transport relevance.

Thus, a fully biocompatible nanovector with ”theranostic” capacities, able to protect active agents from degradation or instability, actively transport and specifically accumulate them at the tumor site, properly modulating their biodistribution, needs be conceived to guarantee the crossing of the BBB and sufficient and selective accumulation in the tumor mass.

Among many biocompatible polymers available for the design of nanovectors, hyaluronic acid (HA) constitutes a promising candidate since it is the principal polymer naturally occurring in the extracellular matrix (ECM) of the brain having a crucial role in glioma invasiveness [26]. Indeed, HA has been found to naturally and directly interact with glioma cells through CD44 receptors which are mediating cell adhesion and invasion [27]. In addition, this natural interaction can be useful to provide an early and more accurate MRI and, in this perspective, it has already been reported that hydrogels, in particular HA, boost relaxometry properties of MRI CAs [28,29]. In detail, Russo et al., demonstrated that HA matrices are able to boost the relaxivity of Gd-based CAs for MRI by a hydrodenticity effect [30,31,32,33]. Indeed, hydrodenticity effect arises from the establishment of a complex equilibrium between the elastodynamics of polymer chains of the hydrogel and water molecules which are packed around Gd-chelates forming gado-meshes that, improving the hydration degree of the CA, boost its relaxivity. Through the hydrodenticity, in particular, they demonstrated that the combination of Gd-DTPA with crosslinked hyaluronic acid nanoparticles (cHANPs) is able to boost its relaxivity up to 12 times.

Because of the aforementioned drawbacks, alternative treatments to TMZ have been widely explored [34]. Among others, the topoisomerase I inhibitor irinotecan (CPT-11) showed encouraging results. Indeed, CPT-11 is FDA approved as adjuvant therapy in colon-rectal cancer and its liposomal formulation ONYVIDE™ was approved by the FDA in 1996 for the treatment of metastatic adenocarcinoma of the pancreas. Recently, both in preclinical test and Phase I clinical trial CPT-11 loaded liposomes (NL-CPT11-NCT00734682) have been tested in patients with recurrent malignant glioma. In 2018, Taghizadehghalehjoughi et al., reported that CPT-11 loaded PLGA nanoparticles administered through convection-enhanced delivery in tumor-bearing mice, produced a significant reduction in tumor volume, much higher than free drug in the same concentration [35].

As stated, further benefits in the delivery of active compounds overcoming biological barriers can be obtained by triggering an active-targeting approach. Among many routes, targeting receptor-mediated transcytosis can be advantageous since it is possible to exploit the numerous transport proteins exposed by the endothelial layer of the BBB that are physiologically involved in the transport of nutrients.

A receptor that has recently been targeted for brain drug delivery is the low-density lipoprotein receptor-related protein-1 (LDLRP-1), a member of the low-density lipoprotein receptor family physiologically involved in the transcytosis of many proteins and peptides and contributing to BBB integrity [36]. Low density lipoprotein receptor related protein-1 (LRP-1) is highly expressed on BBB and overexpressed on glioma cells [36,37,38] and, for this reason, constitutes a fascinating option for dual targeting purposes. In 2008, Demeule et al., designed the peptide angiopep-2 as an optimized substrate for LRP receptors demonstrating high transcytosis capacities on a BBB in-vitro model [39], and the ability to escape P-efflux pumps [40]. In 2012 Xin et al., conjugated angiopep-2 on poly-ethylene glycol and polycaprolactone (PEG-PCL) nanoparticles, confirming in vitro on BECE cells that the transport at the BBB is mediated by LRP transcytosis and in vivo that nanoparticles are able to accumulate in brain parenchyma of healthy mice [41,42].

In this study, we address the limitations of the current diagnosis, treatment and monitoring of GBM, exploiting the principle of hydrodenticity in the design of theranostic crosslinked hyaluronic acid nanoparticles (Thera-cHANPs) through a hydrodynamic flow focusing (HFF) approach obtained by microfluidics. The combination of the dual targeting ability of angiopep-2 with improved Imaging and radiotherapeutic potential of Gd-DTPA is explored in a nanovector able to selectively target glioma cells and increase the cellular uptake of the chemotherapeutic drug irinotecan.

## 2. Results

### 2.1. Synthesis and Chemical-Physical Characterization of Crosslinked Hyaluronic Acid Nanoparticles (cHANPs) and Angiopep-2 Decorated Crosslinked Hyaluronic Acid Nanoparticles (ANG-cHANPs)

**Figure 1:**
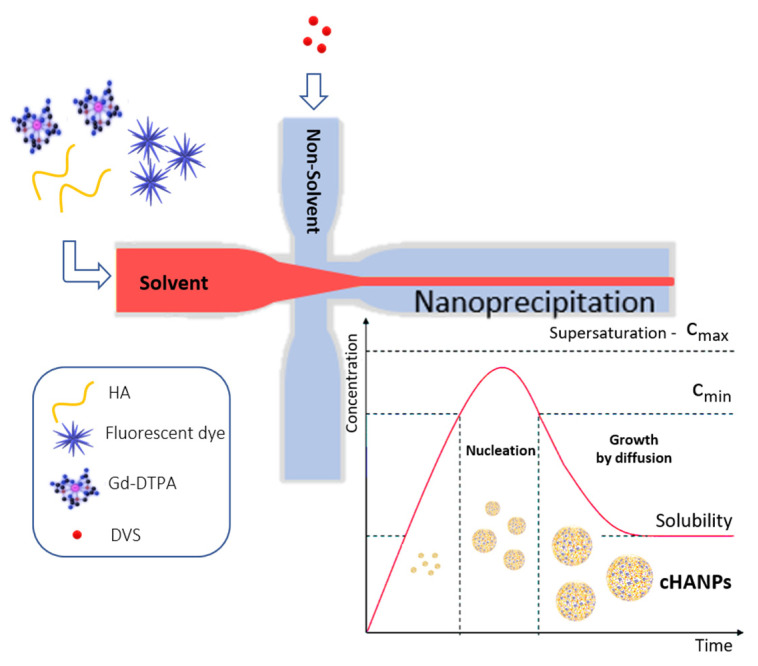
Schematic representation of the microfluidic set-up for crosslinked hyaluronic acid nanoparticles (cHANPs) production.

Crosslinked hyaluronic acid nanoparticles (cHANPs) co-loaded with the metal chelate Gd-DTPA and the fluorophore ATTO 488 are produced in microfluidics by nanoprecipitation through a hydrodynamic flow focusing regime in standard operative conditions as described in the method section and previously reported [29,43]. Briefly, nanoparticles are produced in a microfluidic X-junction chip where the solvent solution of HA and active agents (Gd-DTPA and ATTO 488 or ATTO 633 alternatively) is fed in the middle channel and the non-solvent solution made of acetone and DVS, in the side channels. A schematic representation of the microfluidic set-up for cHANPs production is reported in Figure 1.

Theranostic cHANPs (Thera-cHANPs) co-encapsulating CPT-11 and Gd-DTPA are produced through a cosolvation strategy, adding 10% *V*/*V* of ethanol and 0.025% *w*/*V* of CPT-11 at to the solvent solution of HA and Gd-DTPA because of low CPT-11 water solubility (~1 mg/mL). Solvent solution is injected in the middle channel at 27 µL/min. As in the other experiments, acetone and DVS are fed in the side channels as non-solvent solution at 110 µL/min. A schematic representation of the microfluidic set up for Thera-cHANPs production is reported in Figure S1.

**Figure 2:**
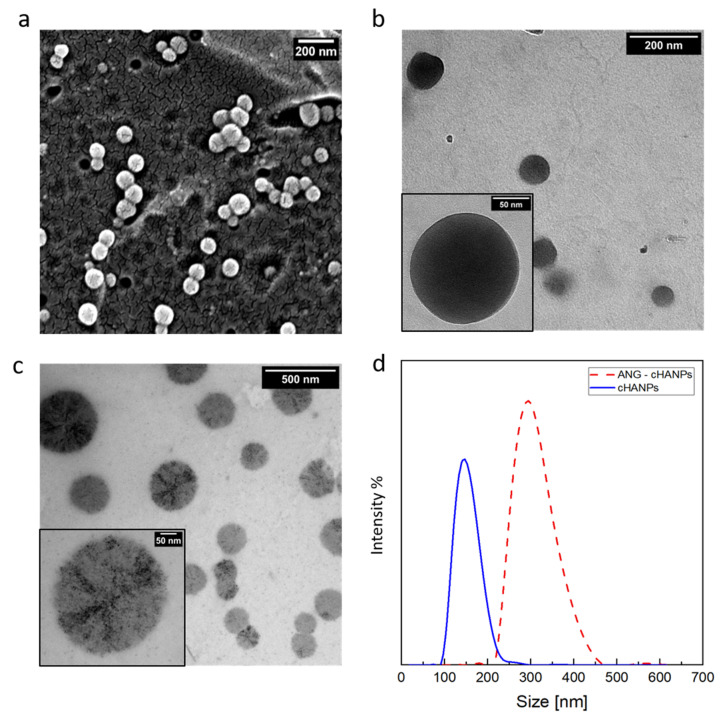
Morphological characterization of crosslinked hyaluronic acid nanoparticles (cHANPs) and angiopep-2 decorated crosslinked hyaluronic acid nanoparticles (ANG-cHANPs). (**a**) SEM images of cHANPs; (**b**) TEM image of cHANPs; (**c**) TEM images of ANG-cHANPs; (**d**) Size distributions of cHANPs and ANG-cHANPs by DLS measurements.

Scanning and transmission electron microscopy (SEM and TEM, respectively) observations of the cHANPs are reported in Figure 2a,b. SEM images and PSD of Thera-cHANPs are shown in supporting Data in Figure S2a,b. In the following steps, nanoparticles are conjugated with the 19 amino acid peptide, angiopep-2, which confers a dual-targeting ability to the nanostructures [41,42]. The procedure is optimized by testing the addition of different concentrations of angiopep-2 and different reaction times (the complete set of data is reported in Figures S3–S5 and Table S1). The addition of 50 µg of peptide per mL of suspension after 4 h of contact, reveals the highest amount of bound peptide (27.63 µg/mL) and an efficiency of the bioconjugation reaction of 55.27%.

To assess the stability of nanoparticles against angiopep-2 conjugation, a morphological analysis on angiopep-2 decorated crosslinked hyaluronic acid nanoparticles (ANG-cHANPs) is conducted by TEM and DLS and a comparison between pre and post bioconjugation is available observing Figure 2b,c, respectively. With respect to cHANPs in Figure 2b, ANG-cHANPs reported in Figure 2c clearly show changes in the morphological structure probably promoted by the peptide, which, during the reaction, is bound not only to the external surface but also partially to the internal hydrogel network. The significant increase in the mean size of nanoparticles pre and post bioconjugation reported in Figure 2d by DLS analysis can be attributed to hydrogel swelling due to bioconjugation. When conjugated to angiopep-2, uncrosslinked polymer chains of nanoparticles are sliding one on the other producing an increase in size. Indeed, the particle size distribution (PSD) shows an increase in the mean diameter of the nanoparticles from about 150 nm to 300 nm after the bioconjugation. It is also important to point out the DLS measurement could reasonably overestimate the hydrodynamic radius of nanoparticles as reported previously by Stetefeld et al. [44]. Indeed, the measured size could be related to the slower diffusion of water layer surrounding the nanoparticles and induced by the extension of the peptide in the continuous medium, leading to increased apparent mean size. This effect has been already reported by Silva et al. [45], who observed for chitosan/alginate nanoparticles a significant mismatch between the hydrodynamic radius measured by DLS and the size of nanoparticles observed at TEM in a dried state. The overestimation of size by DLS has been similarly discussed.

### 2.2. Co-Loading Capability and Stability of cHANPs and ANG-cHANPs with Gadolinium-Diethylenetriamine Pentaacetic Acid (Gd-DTPA), Atto488 and Irinotecan

To study the effect of the conjugation reaction on the encapsulation and stability of payload agents, the encapsulation efficiency (EE%) of both Gd-DTPA, ATTO 488 and irinotecan is calculated for cHANPs, ANG-cHANPs, Thera-cHANPs and Thera-ANG-cHANPs as reported in the dedicated method section. Table 1 summarizes the results showing that all the loaded compounds are retained inside ANG-cHANPs and Thera-ANG-cHANPs with a partial loss of about 50% with respect to cHANPs. The loss of cargo may be attributed to the re-arrangement of the hydrogel network during the bioconjugation reaction that we discussed in the previous section, causing the swelling of the hydrogel, the imbibition of water and diffusion of some molecules out of the hydrogel [46].

**Figure 3:**
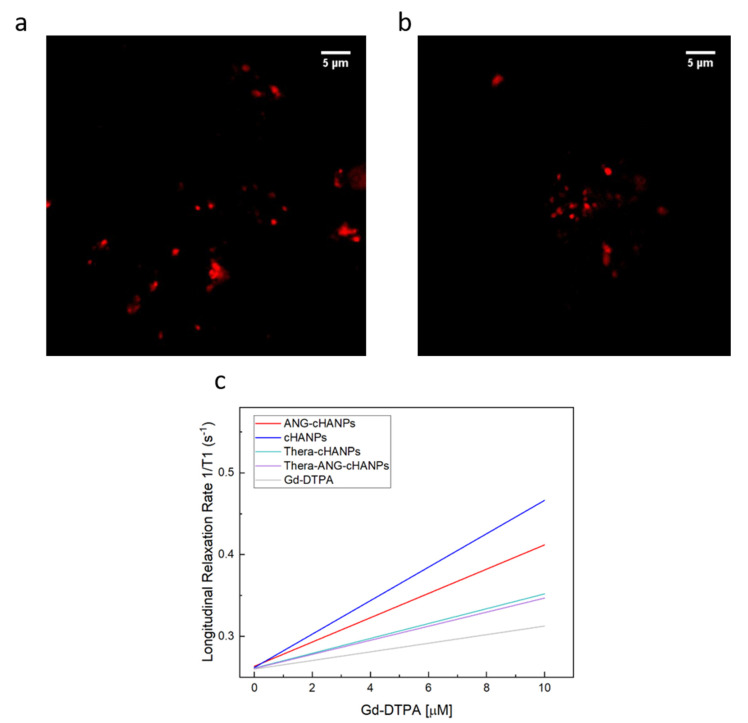
Simultaneous encapsulation of gadolinium-diethylenetriamine pentaacetic acid (Gd-DTPA) and ATTO in cHANPs and ANG-cHANPs. (**a**) Confocal microscopy images of cHANPs; (**b**) Fluorescent microscopy images of ANG-cHANPs; (**c**) Longitudinal relaxation rate (1/T1) in s^−1^ of free Gd-DTPA in water (grey line), cHANPs (blue line), and ANG-cHANPs (red line), Thera-cHANPs (cyan line); Thera-ANG-cHANPs (violet line) showing a relaxivity r1 of 3.58, 20.32, 12.08, 9.09 and 8.86 mM^−1^s^−1^ respectively.

Confocal images of cHANPs and ANG-cHANPs are presented in Figure 3a,b to localize the presence of ATTO 488 inside nanoparticles by the observation of fluorescent spots. Because of the low spatial resolution of the optical light, the dimension of the spots does not correlate with the actual particle size. These observations confirmed that ATTO 488 is retained in ANG-cHANPs.

The ability of hydrogel matrices to boost the relaxivity of Gd-chelates has been widely described by the hydrodenticity theory [29,30,31,32,33,47].

We just reported that the bioconjugation reaction with angiopep-2 promotes a variation in morphology of ANG-cHANPs and a partial loss of co-loaded compounds. In this section, evaluating the relaxivities of cHANPs and ANG-cHANPs, we aim to understand if the aforementioned changes in structural parameters of the polymer network influence the hydrodenticity effect.

Figure 3c presents the relaxation rates in s^−1^ of cHANPs and ANG-cHANPs, both significantly higher than the relaxation rate of free Gd-DTPA. Indeed, cHANPs and ANG-cHANPs show a relaxivity of 20.32 mM^−1^s^−1^ and 12.08 mM^−1^s^−1^, which are respectively 5.67 and 3.38 folds higher than the relaxivity of free Gd-DTPA (3.58 mM^−1^s^−1^). Distributions of the longitudinal relaxation time for both cHANPs and ANG-cHANPs are presented in Figure S6, Tables S2 and S3.

As already reported by Russo et al. [30], the structural parameters of the polymer network that play a major role in tuning the hydrodenticity effect are the crosslinking density and the mesh size of the hydrogel. Therefore, we can hypothesize that the lower relaxivity of ANG-cHANPs may be due to the presence of angiopep-2 in the bulk of nanoparticles and to the rearrangement of the structure, which is affecting the mesh size, thus changing the resulting relaxivity.

Overall, results demonstrate that, despite the establishment of a new equilibrium in the crosslinked matrix of the hydrogel network and the reduced amount of Gd-DTPA in ANG-cHANPs, a significant relaxivity boosting is still present. For this reason, we can confirm that ANG-cHANPs still preserve the hydrodenticity effect.

### 2.3. In-Vitro Studies

#### 2.3.1. Stability of ANG-cHANPs in Culture Medium and Quantitative Uptake by U87 and GS-102 Cells

The uptake of cHANPs and ANG-cHANPs by two human glioblastoma cell lines was quantified by flow cytometry [48]. Firstly, U87-MG cells are used as well-established in vitro model of human glioblastoma and secondly serum-free cultured patient-derived primary glioblastoma stem-like cells (GS-102) were used to confirm results on cells more closely resembling the original tumor molecular profile [49,50,51]. As previously published, serum-free 2D culture on ECM allows attachment of the GS-102 cells preserving cell molecular features and reliable viability assessment. This allows rapid screening of candidate therapies and reliable comparison of efficacy with the 2D culture providing similar results to 3D floating neurospheres [51].

**Figure 4:**
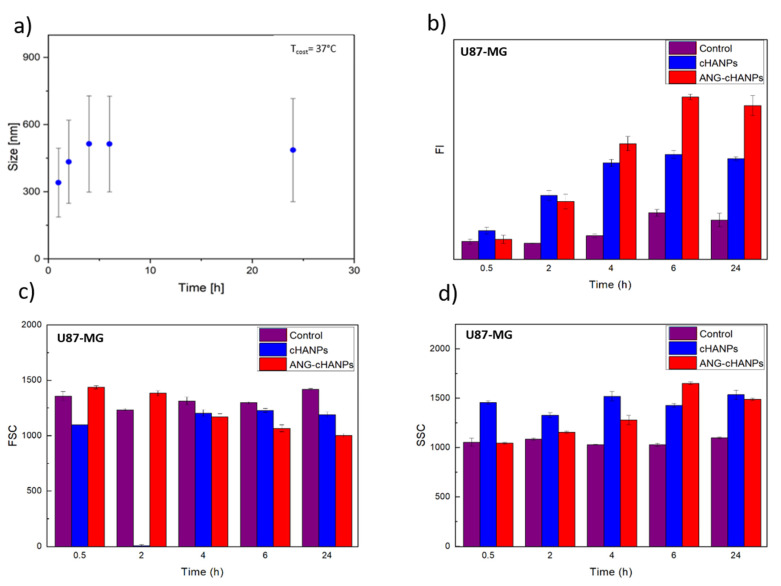
Quantitative uptake of nanoparticles by U87-MG cells in flow cytometry. (**a**) ANG-cHANPs stability in culture medium at 37 °C up to 24 h (**b**) Fluorescence intensity (FI) of U87 after incubation with cHANPs and ANG-cHANPs; (**c**) Forward scattering (FSC) of U87 after incubation with cHANPs and ANG-cHANPs (**d**) Side scattering (SSC) of U87 after incubation with cHANPs and ANG-cHANPs.

As first step, the stability of cHANPs and ANG-cHANPs in culture medium at 37 °C is investigated. Figure 4a reports the variation of the mean size of ANG-cHANPs over time up to 24 h. In the first 4 h, the mean size increases from 300 to 550 nm. This effect can be attributed to the dynamic formation of a protein corona on the nanoparticle surface, increasing the measured hydrodynamic radius [52]. Recently, Yu et al. demonstrated that the protein corona absorbed on hydrophilic nanoparticles undergo quick and frequent exchanges with proteins in solution, displaying a highly dynamic behaviour [53]. This continuous exchange should avoid that the protein corona, masking the nanoparticle surface, affects the interaction of angiopep-2 with cells. The same analysis reported in Figure S7 for cHANPs confirms the stability of nanoparticles in culture medium at 37 °C up to 24 h.

As a consequence, our nanoparticles can preserve their original physicochemical properties in a physiological system, as observed by the in vitro experiments reported in the next figure (Figure 4b–d).

Different studies in literature try to elucidate the effect of size on the internalization of nano/microparticles. However, the attribution of the predominant role in the NP internalization to the complete set of NP characteristics (composition, charge, size and surface modification) is the common finding. Despite this, it has been found that nanoparticles in the range of 200 nm to 1 µM in size (where both cHANPs and ANG-cHANPs lie) are mainly internalized by endocytosis and in particular by micropinocytosis mechanisms [54]. To elucidate about this, cHANPs and ANG-cHANPs are incubated with U87-MG cells and fluorescence intensity (FI), measured in flow cytometry at different time points, is reported in Figure 3b. Both cHANPs and ANG-cHANPs display a significantly higher fluorescence than control at any time point. For short times (i.e., up to 4 h), a linear increase in fluorescence is observed, with ANG-cHANPs showing a higher slope (linear fit in Figure S8). Salvati et al. [55] reported that a linear increase in fluorescence is typical of energy-dependent endocytic uptake of nanoparticles characterized by stable fluorescent labeling. Indeed, in the presence of labile dye, a sudden increase in fluorescence and a sharp drop in the very first hours would be observed. After 4 h of contact, cHANPs fluorescence reaches a plateau, remaining almost unmodified up to 24 h. In the same work, Salvati et al. identified this plateau as an equilibrium condition in which the internalization process saturates and nanoparticles reach lysosomes [55]. On the other hand, ANG-cHANPs fluorescence continuously increases, indicating a different uptake mechanism, which is not saturated in the first 4 h of contact. This observation is in accordance with Bertrand et al. [40] who reported that the uptake mechanism of angiopep-2 is a receptor-mediated transcytosis by LRP-1 receptors. In addition, the difference in the slope of the linear uptake of the very first hours, confirms a difference in the uptake rate, thus in the underlying uptake mechanism, which is much faster for ANG-cHANPs than cHANPs. Both for cHANPs and ANG-cHANPs at 24 h, a slight decrease in fluorescence intensity can be observed. This phenomenon can be attributed to cell division and thus nanoparticle redistribution in daughter cells [48].

All the observations are supported by FSC and SSC measurements reported in Figure 4c,d, respectively. FSC decreases over time and SSC increases with the same trend observed in fluorescence intensity. These effects are well described by Jochums et al. [56] who reported that nanoparticles located inside the cell, similarly to granulocytes, generate an increase in the SSC intensity in a dose-dependent manner so that the addition of nanoparticles to the cytoplasm can cause increased SSC and decreased FSC.

**Figure 5:**
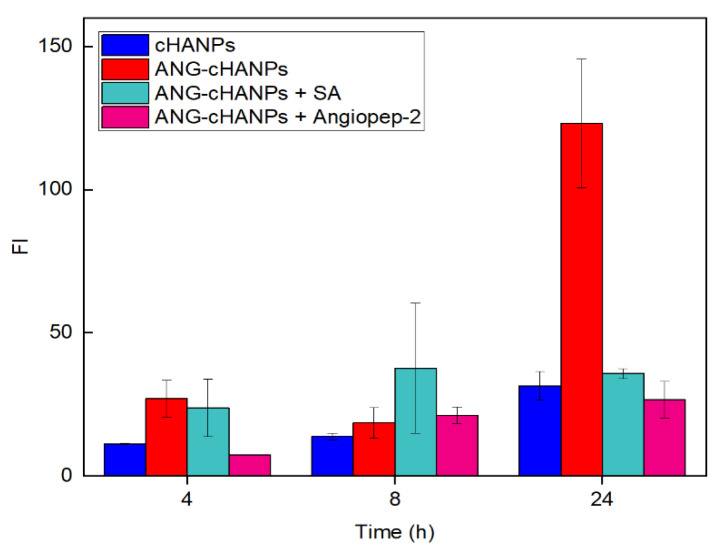
Fluorescent Intensity (FI) of U87 cells after co-incubation with ANG-cHANPs and sodium azide (SA) to deplete cellular ATP, and excess of angiopep-2 for the competitive binding of low density lipoprotein receptor related protein-1 (LRP-1) receptors.

To confirm the involvement of angiopep-2 and of an energy-dependent mechanism in the uptake of ANG-cHANPs, we quantified ANG-cHANPs internalization in ATP-depleted cells and in competition with excess of angiopep-2. Internalization was quantified by measuring single cell fluorescent intensity by flow cytometry at 4 h, 8 h and 24 h of incubation. Results in Figure 5, as expected, reveal that the depletion of ATP and the treatment of cells with angiopep-2 caused a decrease in Fl intensity with respect to untreated cells, due to less internalized nanoparticles. Already at 4 h of incubation, both in ATP-depleted cells and angiopep-2 treated cells the fluorescence intensity is strongly reduced and at 24 h and this reduction reaches 71% and 82%, respectively. These results confirm the involvement of both an energy dependent-mechanism and the peptide angiopep-2 in the internalization of ANG-cHANPs.

**Figure 6:**
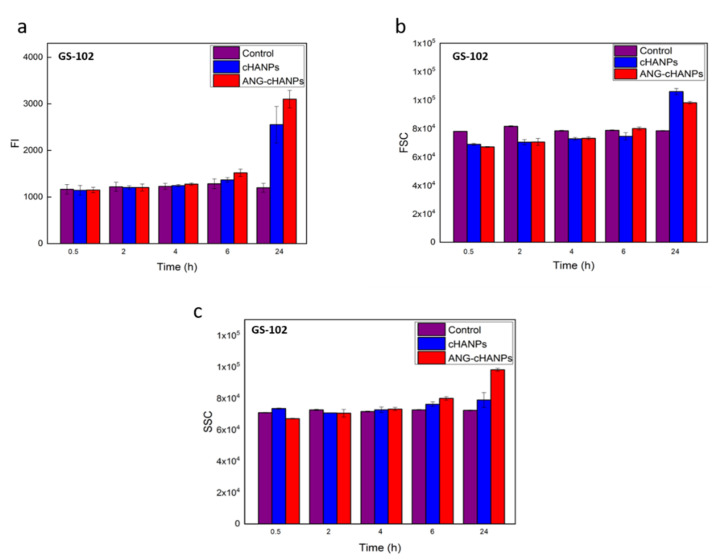
Quantitave uptake of NPs by GS-102 cells in flow cytometry. (**a**) Fluorescence intensity (FI) of GS-102 after incubation with cHANP and ANG-cHANPs; (**b**) Forward scattering (FSC) of GS-102 cells after incubation with cHANPs and ANG-cHANPs (**c**) Side scattering (SSC) of GS-102 cells after incubation with cHANPs and ANG-cHANPs.

The study of NPs uptake by flow cytometry was then conducted on patient-derived GS-102 cells and FI in Figure 6a reveals that a significant uptake both of cHANPs and ANG-cHANPs can be observed with a delay of 6 h with respect to U87-MG cells. Despite this time lag, at 24 h an exponential increase in fluorescence, more pronounced for ANG-cHANPs than cHANPs, is present. These results, which are in accordance with SSC and FSC measurements presented in Figure 6b,c respectively, confirm the involvement of a different mechanism of uptake for cHANPs and ANG-cHANPs.

#### 2.3.2. ANG-cHANPs Localization in U87 Cells

**Figure 7:**
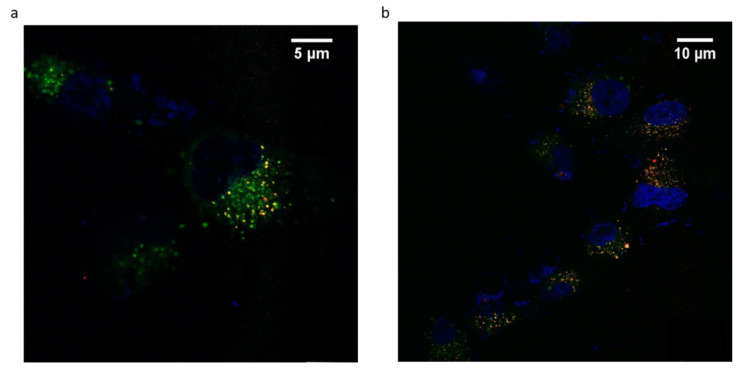
ANG-cHANPs internalization by U87-MG cells at a concentration of 50 µg/mL. (**a**) Time-dependent nanoparticle localization in the cell by confocal microscopy after (**a**) 4 h of incubation; (**b**) 6 h of incubation. Blu: Hoechst 33342, nuclei; Green: Lysotracker Green, lysosomes; Red: ATTO633, ANG-cHANPs.

ANG-cHANPs localization in U87-MG cells is studied by confocal imaging. This study is performed with ANG-cHANPs encapsulating Gd-DTPA and ATTO 633 (EE% 10%), a NIR dye, in place of ATTO 488 in order to avoid artifacts due to the strong autofluorescence of biological samples in the visible spectrum [57]. Figure 7a,b confirm ANG-cHANPs uptake, showing time-dependent nanoparticle accumulation into cells. Both images confirm the stability of ATTO 633 loading inside nanoparticles considering the absence of diffuse fluorescence associated with free dye [55]. As previously observed by flow cytometry results reported in Figure 4b–d, Figure 7a confirms a relevant uptake of ANG-cHANPs after 4 h of incubation, also revealing a partial correlation of nanoparticles with lysosomes identified by yellow spots (the overlap of red and green fluorescence of ANG-cHANPs and lysosomes respectively). In Figure 7b after 6 h of incubation, a stronger correlation of ANG-cHANPs with lysosomes can be observed. These results perfectly match the flow cytometry results.

#### 2.3.3. Thera-cHANPs and Thera-ANG-cHANPs Uptake in U87 Cells

**Figure 8:**
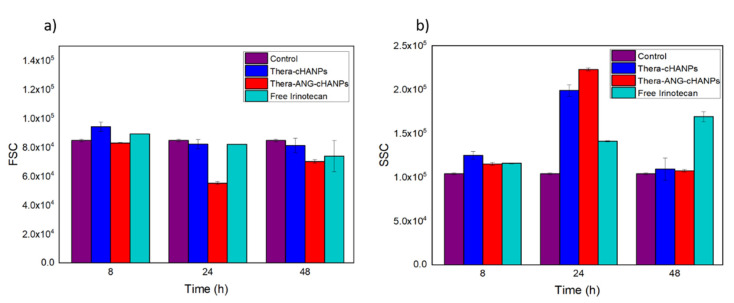
Quantitative uptake of NPs by U87-MG cells by flow cytometry. (**a**) Forward scattering (FSC) of U87 after incubation with theranostic Thera-cHANPs and Thera-ANG-cHANPs (**b**) Side scattering (SSC) of U87 after incubation with Thera-cHANPs and Thera ANG-cHANPs.

Flow cytometry measurements are performed on U87 MG cells incubated with free CPT-11, Thera-cHANPs and Thera-ANG-cHANPs at the same concentration of CPT-11 of 10 µM at different time points. The FSC measurement was used as an indication of cell viability [56,58], with the aim to assess the therapeutic potential of the nanoformulations and compare it to the free drug. As CPT-11 is a topoisomerase inhibitor acting on cell division, three time points up to 48 h were analysed to allow all the cells to complete the cell cycle. Similar to the previous experiment, the measurements of SSC were used to evaluate the amount of internalized nanoparticles, being an indication of the granularity of the cells. In this way, both the role of the nanovector itself and angiopep-2 in improving the uptake of CPT-11 can be evaluated. Figure 8a shows that starting from 24 h, the FSC both for Thera-cHANPs and Thera-ANG-cHANPs is decreased with respect to the control. In particular, the FSC of Thera-ANG-cHANPs is significantly decreased of about 35% at 24 h. The increase in FSC at 48 h may be attributed to remaining live cells that continue to replicate, with internalized NPs dividing in daughter cells causing a decrease in the amount of CPT-11 per cell. This hypothesis is supported by the significantly decreased SSC value. FSC values of free CPT-11 are higher than nanoparticles up to 48 h showing that free CPT-11 has a delayed effect with respect to nanoformulated CPT-11, which confirms improved uptake capacity of Thera-ANG-cHANPs. SSC values in Figure 8b confirm these results, which are significantly higher for Thera-cHANPs and Thera-ANG-cHANPs up to 24 h. Also in this case, free CPT-11 reaches the highest internalization only at 48 h. Both FSC and SSC confirm the improved uptake of nanoformulated CPT-11 which is significantly faster with respect to free drug. This effect is particularly evident for Thera-ANG-cHANPs.

To further confirm the therapeutic potential of the formulations, an MTT assay is performed on U87 cells after incubation with 10 µM of free irinotecan, irinotecan in Thera-cHANPs, and in Thera-ANG-cHANPs at 24 h and 48 h. Results presented in Supplementary data file in Figure S9, confirm the improved therapeutic effect of Thera-ANG-cHANPs over the other treatments at the same concentration of drug. Importantly, the effect of Thera-ANG-cHANPs is already evident at 24 h, where the cell viability is reduced to 62%, while free irinotecan causes a reduction only at 92%. These results match flow cytometry measurements.

## 3. Discussion

The challenge of effectively delivering therapeutic agents to the brain has created an entire field of active research devoted to overcoming the blood-brain barrier (BBB) and efficiently delivering drugs to the brain. We proved that theranostic-angiopep-2 hyaluronic acid nanoparticles (Thera-ANG-cHANPs) with improved relaxometric and therapeutic properties and uptake capacities potentially promise a theranostic application to glioma treatment.

Multimodal crosslinked hyaluronic acid nanoparticles (cHANPs) co-loaded with the metal chelate Gd-DTPA and the fluorophore ATTO 488 are produced by nanoprecipitation through a microfluidic hydrodynamic flow focusing regime. Microfluidics has shown to be tremendously promising in nanomaterial translation to clinics in the last years, demonstrating advanced nanomaterial characteristics’ controllability and uniformity [59]. Indeed, we also showed how it could be implemented to finely tune structural changes in hydrogels’ formation to induce the hydrodenticity and boost T1 MRI relaxivity of Gd-based CAs [29,30,33,43].

Moreover, through microfluidics, the theranostic properties are achieved by co-encapsulating CPT-11 (irinotecan) and Gd-DTPA, simultaneously through a one-step co-solvation strategy producing the Thera-cHANPs. Irinotecan has been selected because it is emerging as a valid alternative to the wide-spread used temozolomide that is the primary drug applied in the first-line treatment of glioma. CPT-11 is an inhibitor of topoisomerase I; it is FDA approved to treat metastatic adenocarcinoma of the pancreas and, currently, repositioned and tested in Phase I clinical trials for the treatment of malignant glioma.

Finally, delivery of the Thera-cHANPs is reached by using angiopep-2 able to trigger transcytosis and traverse the BBB by recognizing low-density lipoprotein related protein-1 (LRP-1) expressed on the brain capillary endothelial cells and overexpressed on glioma cells. angiopep-2 is currently in clinical trials in combination with several nanomedical formulations for brain delivery. Our in vitro tests on Thera-ANG-cHANPs using U87-MG cells and serum-free cultured patient-derived primary glioblastoma stem-like cells (GS-102) demonstrated selective uptake capacities of the Thera-ANG-cHANPs, and the ability to escape P-efflux pumps, even at cellular conditions closer to the original molecular profile. These results are obtained by preserving the hydrodenticity and the loading ability of the nanocarrier.

Thera-ANG-cHANPs proposed rational design is inspired by the need to improve the delivery efficiency of the nanovectors and obtain a strong correlation between the synthetic identity of the nanocarrier and the requirements to overcome biological barriers. Indeed, the specific design of nanoparticle-based formulations can play a role in controlling biodistribution, transport properties and mechanisms of the active agents for therapy and diagnosis [6,24,60]. However, although efficient delivery and distribution of treatment agents over the whole tumor are essential for successful tumor treatment, most of these agents’ distribution cannot be visualized or the efficacy of the treatment cannot be recognized. This is particularly true for the glioma, where the diffusion of active molecules happens only into the perinecrotic regions of the tumor mass characterized by newly formed and leaky blood vessels [61].

In this framework, GBM diagnosis, treatment, and monitoring are significantly delayed depending on the tumor’s vascularization and may exclude a significant portion of the total mass. Our Thera-ANG-cHANPs design is proposing a strategy based on precision medicine, implementing simultaneously dual targeting approach by angiopep-2 and the hydrodenticity effect for evaluating the therapeutic efficacy of a recent repositioned drug for theranostic purposes. The proposed nanoformulations can serve as a valuable tool for selecting a safe and effective dose, recognizing adverse effects, and real-time objective monitoring. Indeed, the boosted MRI provided by the nanoparticles based on hydrodenticity could be used to better understand the ‘exchange-related’ DCE-MRI-derived parameters to elucidate the relation between vascular characteristics, drug delivery and treatment efficacy. The proposed theranostic approach providing the combination of improved molecular imaging functionalities with therapy may contribute significantly to the growing field of personalized medicine.

## 4. Materials and Methods

Sodium hyaluronate (M_w_ = 42,000 Da) was purchased from Bohus Biotech (Strömstad, Sweden). Diethylenetriaminepentaacetic acid gadolinium(III) dihydrogen salt hydrate Gd-DTPA (M_w_ = 547.57 Da), acetone (CHROMASOLV, for HPLC, ≥99.8%; molecular formula CH_3_COCH_3_; M_w_ = 58.08 Da), ethanol (ACS reagent, ≥99.5% (200 proof), absolute; molecular formula CH_3_CH2OH; M_w_ = 46.07 Da), divinyl sulfone (DVS) (contains <650 p.p.m. hydroquinone as inhibitor; purity 97%; density 1.117 g/mL at 25°C (lit.) molecular formula C4H6O2S, M_w_ = 118.15 Da), sodium hydroxide NaOH (ACS reagent, ≥97.0%, M_w_ = 40.00 Da), ATTO 488 (λex/em= 488/560 nm, M_w_ = 804 Da) ATTO 633 (λex/em= 633/670 nm, M_w_ = 652 Da), (3-dimethylaminopropyl)-N′-ethylcarbodiimide hydrochloride EDC (molecular formula C8H17N3∙HCl; M_w_ = 191,70 Da), N-hydroxysuccinimide NHS (molecular formula C_4_H_5_NO_3_; M_w_=115,09 Da) were all purchased from Merck KGaA (Darmstadt, Germany). Angiopep-2 (TFFYGGSRGKRNNFKTEEY, M_w_ = 2625.8 Da) was purchased from ProteoGenix SAS (Schiltigheim, France). QuantiPro™ BCA Assay Kit was purchased from Sigma Aldrich (St. Louis, MO, USA). The human glioblastoma cell line U87 (passage 15–36) was purchased from ATCC (Manassas, VA, USA). Dulbecco Modified Eagle Medium high glucose (DMEM), fetal bovine serum (FBS), phosphate buffer saline (PBS) and human serum albumin (HSA) for cell culture and in-vitro studies were purchased from from Sigma Aldrich Co. (St. Louis, MO, USA). The water used for synthesis and characterization, was purified by distillation, deionization, reverse osmosis (Milli-Q Plus, Q-POD^®^, Merck KGaA, Darmstadt, Germany) and finally filtered with a 0,22 μm cutoff filter.

### 4.1. Preparation of Co-Loaded cHANPs through the Microfluidic Platform

Microfluidic production of crosslinked hyaluronic acid nanoparticles (cHANPs) encapsulating both Gd-DTPA and a fluorophore has been widely described in our previous works [29,30,43]. Briefly, nanoparticles are produced in a microfluidic X-junction chip where the hydrodynamic flow focusing regime is used to implement a nanoprecipitation process. The solvent solution of HA and active agents is fed in the middle channel and the non-solvent solution made of acetone and DVS, is fed in the side channels. In this work, two fluorophores, ATTO 488 and ATTO 633, and the chemotherapeutic drug Irinotecan are alternatively encapsulated to investigate further the chemical-physical, the biological abilities and the theranostic potential of the cHANPs and ANG-cHANPs. The solvent solution is a water solution of HA (0.05% *w*/*V*), Gd-DTPA (0.1% *w*/*V*) and fluorophore (10 nmol/mL). To encapsulate irinotecan, a co-solvation strategy is explored. The solvent solution is made of HA (0.05% *w*/*V*), Gd-DTPA (0.1% *w*/*V*), Irinotecan (0,0025% *w*/*V*) and ethanol 10% *V*/*V*. In all cases solvent solution is fed in the middle channel by a 2.5 mL glass syringe at 27 µL/min. The non-solvent solution of acetone and DVS (4 M) is fed in the side channels by two glass syringes of 10 mL each at 110 µL/min. The pH of the solvent solution is adjusted at 12.2 by NaOH addition to promote the occurrence of the crosslinking reaction between HA and DVS. The sample is collected in 20 mL of acetone and well stirred overnight to promote DVS diffusion and complete the crosslinking reaction. Purification is performed by two-step solvent gradient dialysis, dropping the suspension in Spectra Por Cellulose Membrane 6 (MWCO 50 kDa, Sigma Aldrich Co., St. Louis, MO, USA) The first purification step is in ethanol and the second in water, through an increasing gradient of acetone-ethanol and ethanol-water respectively (70%/30% *V*/*V*, 50%/50%*V*/*V*, 30%/70%*V*/*V*, 100% in both steps). Diffusion is promoted by continuous stirring at 230 rpm and room temperature.

### 4.2. Synthesis of Co-Loaded ANG-cHANPs

Angiopep-2 is conjugated to nanoparticle surface through the amidation reaction of free carboxyl groups of cHANPs and amine groups of angiopep-2. 500 µL of cHANPs suspension containing 5 × 10^8^/mL NPs, is added to an aqueous solution of EDC (0.02 M) and NHS (0.006 M) and let react for 10 min under continuous wheel stirring to activate available carboxyl groups. Then, angiopep-2 (50 µg/mL) is added and the suspension is continuously wheel stirred prior purification.

The procedure is optimized by testing the addition of different concentrations of angiopep-2 and different contact times to maximize the efficiency of the reaction. The purification from unreacted peptide is performed either by ultracentrifugation (UC) or by centrifugation in Corning^®^ Spin-X^®^ UF Concentrators (CC) with a MWCO of 30 kDa or 50 kDa by Corning (Ithaca, NY, USA).

The quantitative BCA assay is used to measure the amount of peptide successfully bound to cHANPs. Typically, 150 µL of sample and 150 µL of buffer solution are put in a 96 well-plate, and let react for 1 h at 60 °C. Therefore, the absorbance of purified samples is measured at 562 nm and the amount peptide in the sample quantified through a calibration curve (0.5–30 µg/mL), Figure S10. The absorbance of bare cHANPs (7,5 × 10^7^ NPs/mL) is measured for each batch and subtracted to the absorbance of ANG-cHANPs at the same concentration prior to quantification.

Triplicates of different dilutions of the samples (1:2, 1:5, 1:10) are measured in order to confirm the repeatability of the measurement and to average the effect of measuring a suspension that is likely subjected to inter-measurement variability. The efficiency of the conjugation reaction is calculated dividing the measured amount of peptide in the purified sample by the one added initially.

### 4.3. Chemical-Physical Characterization of cHANPs and ANG-cHANPs

Size, polydispersity, surface charge and stability in culture medium of the bare and conjugated NPs are investigated by dynamic light scattering (DLS) by Zetasizer Nano, Malvern Pananalytical (Malvern, UK). The size measurement is performed at 173 and Room Temperature. 1 mL of suspension is dropped in a square glass cuvette (Optical Cuvette, Sarstedt, Verona, Italy) and measured in triplicate. In the work, size distribution measured by DLS is reported as Particle Size Distribution (PSD).

The stability of the suspension in culture medium (DMEM, 1% penicillin/streptomycin and 1% L-glutamine) is studied by DLS measurements at 37 °C. Typically, 10 µL of suspension are dropped in 1 mL of culture medium and triplicate measurements are automatically acquired every hour up to 24 h. Zeta potential measurements are performed at Room Temperature on a Zetasizer Nano (Malvern Pananalytical, Malvern, UK), filling 1 mL in a high-concentration zeta potential cell.

The morphology of nanoparticles is studied by scanning electron microscopy (SEM, Ultraplus Field Emission, Carl Zeiss, Oberkochen, Germany) and transmission electron microscopy (TEM), Cryo-TEM TECNAI, FEI (Hillsboro, OR, USA).

Samples for SEM observations are prepared by depositing 200 µL of suspension (5 × 10^8^ NPs) by ultrafiltration on polycarbonate Isopore^TM^ membrane filters with a 50 nm cut-off form Merck KGaA (Darmstadt, Germany) then let dry. On the membrane filter 7 nm Au are deposited via sputter coating and observed at 10 kV. TEM samples are prepared by dropping 20 µL of suspension (5 × 10^7^ NPs) on copper grids with carbon film by Agar Scientific Ltd. (Stansted, UK) and let dry prior observation at 120 kV.

### 4.4. Quantitative Analysis of Co-Loaded Gd-DTPA, ATTO and Irinotecan in NPs

To quantify the content of Gd-DTPA in NPs, Inductively Coupled Plasma ICP-MS NexION 350 measurements, PerkinElmer Inc. (Waltham, MA, USA), are performed. The software interface Syngistix NanoApplication (Module PerkinElmer Inc., Waltham, MA, USA) is used to run the experiment and collect all data. In the Gd-DTPA measurement, *m*/*z* 157, 100 μs dwell time and no settling time are set.

Determination of Irinotecan, ATTO488 or ATTO633 content is performed through a Multiplate Reader Photometer Enspire Perkin-Elmer Inc (Waltham, MA, USA) (Abs at 368 nm, λ ex/em 488–560 and 633–670 nm, respectively). The calibration curve to correlate absorbance and fluorescent intensity to drug and fluorophore concentration is obtained, Figure S11. For Gd-DTPA, Irinotecan and the fluorophore, the encapsulation efficiency (EE%) is calculated as:

$$EE\%=\;\frac{C_{M}}{C_{I}}∙100,$$

where $C_{M}$ is the measured amount of substance in the purified sample and $C_{I}$ its theoretical concentration which is the concentration calculated considering the amount of solute injected in the collection volume.

Fluorescent imaging technique by SP5 Laser Scanning Confocal Microscope, Mannheim, Germany) is used to localize the fluorophore inside nanoparticles by individuation of fluorescent spots, and to confirm the ability of ANG-cHANPs to retain the fluorophore after the conjugation reaction. Nanoparticles are observed with a 60× oil objective and with a HeNe 633 nm laser both in solution in a Willco-dish glass 35 × 10 mm and deposited by ultrafiltration on a polycarbonate Isopore membrane (50 nm cut-off, Merck KGaA (Darmstadt, Germany).

### 4.5. cHANPs, ANG-cHANPs and Thera-ANG-cHANPs Relaxometric Properties

To characterize NPs relaxometric properties, in vitro MR measurements on both co-loaded cHANPs and ANG-cHANPs are performed and results are compared to Gd-DTPA solutions at similar concentrations. After vigorous stirring, 300 μL of the sample (3 × 10^8^ NPs) is dropped in a glass tubes and changes in longitudinal relaxation time (T1) at 1.5 Tesla and 37 °C by Minispec Bench Top Relaxometer (Bruker Corporation, Billerica, MA, USA) are measured. The relaxation time distribution is obtained by a CONTIN Algorithm and the relaxation spectrum is normalized by its processing parameters. The integral of a peak corresponds to the contribution of the measured species to the relaxation time spectrum. Longitudinal relaxation time (T1) is measured both for cHANPs and ANG-cHANPs at different dilutions and the longitudinal relaxation rates in s^−1^ (i.e., the reciprocals of the mean T) are plotted against corresponding Gd-DTPA content, previously determined by ICP-MS measurements. These values are linearly fitted and the relaxivity is obtained in mM^−1^s^−1^ as the slope of the line.

MRI images of nanoparticles were acquired on a 1T SPECT/MRI system (NanoScan; Budapest, Hungary) equipped with a solenoid coil with 35 mm diameter in 30 min. Multiple T1-based images were acquired using a spin-echo sequence (repetition times 550, 880, 1400, 2000 ms, echo time 80 ms) and T1 maps were calculated using MLE method to fit saturation recovery curves (a*exp(−TR/T1) + c). The other scan parameters included an 80-mm field of view, a 96 × 96 matrix, and 2 mm slice thickness.

### 4.6. Flow Cytometry Measurements

Nanoparticle uptake by human brain glioblastoma astrocytoma cells U87-MG and patient-derived primary glioblastoma cells, and their therapeutic potential are studied and a comparison between cHANPs, ANG-cHANPs, Thera-cHANPs and Thera-ANG-cHANPs is performed.

To quantitatively measure internalized cHANPs and ANG-cHANPs after incubation with live cells, flow cytometry analysis (Partec CyFlow Space, Sysmex^®^, Milan, Italy) is performed. A 488 nm wavelength laser is used to excite NPs and fluorescence is collected using a 595–660 nm channel.

Results are reported as the mean of the distribution of FI and FSC and SSC values obtained by measuring a population of 10,000 events averaged between three independent replicas. Error bars correspond to the standard deviation between replicas. Data analysis was performed using CytoFlow software (v 1.0, Massachusetts Institute of Technology 2015-2018, Cambridge, Massachusetts, USA). To investigate the therapeutic potential of Thera-cHANPs and Thera-ANG-cHANPs, live cells are incubated with NPs and Forward Scattering (FSC) and Side Scattering (SSC) are recorded as indication of cell viability [48,55]. Nanoparticles located inside the cell, similarly to granulocytes, will increase the SSC intensity in a dose-dependent manner, so that the addition of nanoparticles to the cytoplasm can cause increased SSC and decreased FSC. This will occur similarly to what has been found for granulocytes inside cells and it could also explain the increase after long incubation times, when the nanoparticles concentration per cell gets lower by cell division. For these reasons, by comparing SSC values with the control (untreated cells), when an increase is detected, nanoparticle uptake is supposed, an example of raw data is presented in Figure S12. This observation is supported by the increase in cell fluorescence due to the uptake of fluorescent nanoparticles. In addition, FSC being an indication of cell area, can be used to reveal apoptosis. Indeed, in the initial stages of apoptosis, the cell shrinks while the membrane remains intact. As a consequence of this, the FSC decreases while the SSC intensity increases or remains unchanged. For this reason, the decreased FSC of nanoparticle treated- or drug treated-cells is used as indication of cell apoptosis (reduced cell area). This data is supported by a decrease SSC indication of nanoparticle/drug internalization [48,55]. 

To elucidate about internalization mechanism of ANG-cHANPs, cells were pre-treated with sodium azide (SA) (1 mg/mL for 30 min) and then co-incubated with ANG-cHANPs (5 × 10^7^ NPs/mL) and SA at the same concentration, to deplete cellular ATP and inhibit energy dependent uptake mechanisms. In a separate experiment, cells were pre-treated with an excess of free angiopep-2 (200 µg/mL for 30 min) and then co-incubated with nanoparticles (5 × 10^7^ NPs/mL) and free angiopep-2 at the same concentration as a competitor for LRP-1 binding. Internalization was quantified by measuring single cell fluorescent intensity by flow cytometry at 4 h, 8 h and 24 h of incubation.

In case of U87-MG, cells were seeded in 12-well plates at a density of 2 × 10^5^ cells/well and grown (Medical Ethical Review Committee Erasmus MC: MEC-2013-090) in DMEM, 10% FBS, 1% penicillin/streptomycin and 1% L-glutamine. After 24 h, cell culture medium supplied with cHANPs or ANG-cHANPs at 5 × 10^7^ NPs/mL was added.

The use of patient tumour material for cell culture establishment was approved by the institutional review board of the ErasmusMC and obtained with patient’s informed consent. Cell cultures were established and cultured in serum-free Dulbecco’s modified Eagle’s medium (DMEM)–F12 with 1% penicillin/streptomycin, B27 (Invitrogen, Waltham, MA, USA), human epidermal growth factor (EGF; 5 μg/mL), human basic fibroblast growth factor (FGF; 5 μg/mL) (both from Tebu-Bio, Le Perray-en-Yvelines, France), and heparin (5 mg/mL; by Sigma-Aldrich., St. Louis, MO, USA) as described previously [51]. For the experiments, patient-derived GS-102 cells which are MGMT methylated cells were seeded in 96-well plates previously coated with growth factor reduced extracellular matrix (ECM), at a density of 5 × 10^4^ cells/well. After 24 h, cell culture medium supplied with cHANPs or ANG-cHANPs at 50 µg/mL was added.

Flow cytometry measurements are performed after 30 min, 2, 4, 6 and 24 h of incubation. At each time point, the medium is removed, and three washing steps with PBS (1×) are carried out to ensure the removal of non-internalized particles. Cells seeded at the same density but supplied with normal culture medium (DMEM, 1% penicillin/streptomycin and 1% L-glutamine, 10% FBS for U87-MG and (DMEM)–F12 with 1% penicillin/streptomycin, B27, EGF (5 μg/mL), FGF (5 μg/mL), and heparin (5 mg/mL) for GS-102) are used as negative controls. Cells are collected by trypsinization and each flow cytometry analysis is conducted at least in triplicate.

### 4.7. Confocal Imaging Analyses

Confocal imaging is performed to study ANG-cHANPs cellular localization. Cells are seeded at a density of 5 × 10^4^ cells/well in 8 slide µ-well glass bottom (IBIDI^®^ GMBH, Gräfelfing, Germany). After 24 h, medium supplied with ANG-cHANPs (150 µg/mL) is added and incubated for 4 h and 6 h. To stain cell nucleus and lysosomes, Hoechst 33342 (0.5 µM) and Lysotracker green (0.1 µM) are added to the culture medium and incubated 15 min. Live cells are observed at Leica Microsystems TCS SP5 Laser Scanning Confocal Microscope (Wetzlar, Germany) equipped with an incubator to keep the temperature at 37 °C and CO_2_ levels at 5%, with a 60× oil objective. Hoechst 33342 is excited with a 405 diode laser, Lysotracker green with an Argon laser at 488 nm and ANG-cHANPs are excited with the HeNe 633 nm laser.

## 5. Conclusions

In this work, Thera-ANG-cHANPs, a fully biocompatible and theranostic nanovector with boosted relaxometric properties and selective uptake capacities of human glioblastoma cells, were proposed for early glioblastoma diagnosis, effective therapy and monitoring.

The design paradigm is made from a physical and a biological perspective to overcome barriers encountered in the body and avoid the off-target site. Indeed, the use of hyaluronic acid supports natural nanoparticle diffusion through the brain ECM and the control of its structural parameters enables the boosting of the relaxometric signal of Gd-DTPA up to 3, 5 times; the effect of the hydrodenticity contributes to an early and accurate diagnosis using an already clinically approved CAs; moreover, the selective transport at subcellular level is obtained by the angiopep-2 through a specific energy-dependent mechanism. Finally, due to this characteristic, the vector was shown to accelerate the therapeutic effect of CPT-11 with an evident cytotoxic effect within 24 h.

The proposed strategy has been conceived by identifying relationships between nanoparticle design and in vivo transport at the organ level, sub-organ level, and subcellular level, and then organizing these relationships into a theranostic system including also clinically relevant features to improve the clinical outcome of glioblastoma treatment.

## Figures and Tables

**Table 1:** Co-loaded cHANPs, ANG-cHANPs Thera-cHANPs and Thera ANG-cHANPs characterization.

	Particle Size [nm]	Zeta Potential [mV]	Gd-DTPA [µM]	Gd-DTPA EE%	ATTO 488 [µM]	ATTO 488 EE%	Irinotecan [µM]	Irinotecan EE%
cHANPs	149.99 ± 29.8	−33.8 ± 4.25	12.02	3.59	0.26	16.5	-	-
ANG-cHANPs	305.6 ± 60.7	−36.3 ± 3.45	5.3	-	0.152	-	-	-
Thera-cHANPs	106.79 ± 46.33	−15.4 ± 6.96	8.11	2.67	-	-	155.46	19.43
Thera-ANG-cHANPs	362 ± 48.40	−7.14 ± 6.05	2.64	-	-	-	65.85	-

Crosslinked hyaluronic acid nanoparticles (*cHANPs*); angiopep-2 decorated crosslinked hyaluronic acid nanoparticles (*ANG-cHANPs*); Theranostic—Crosslinked hyaluronic acid nanoparticles (*Thera-cHANPs*); Theranostic—angiopep-2 decorated crosslinked hyaluronic acid nanoparticles (*Thera-ANG-cHANPs*).

## Data Availability

The data presented in this study are available in this article and supplementary material.

## Supplementary Materials

The following are available online at https://www.mdpi.com/2072-6694/13/3/503/s1. Supplementary figures and tables are provided in the supplementary data file. Figure S1: Schematic representation of Microfluidic experimental set-up for Thera-cHANPs production, Figure S2: SEM images of (a) Thera-cHANPs; (b) PSD of Thera-cHANPs; c) concentrated Thera-cHANPs, Table S1: Different Concentrations of angiopep-2 and related reaction efficiencies, Figure S3: DLS measurements of sediment and supernatant post ultracentrifugation (a) 50000 RPM for 15 min; (b) 70000 RPM for 15 min, Figure S4: Effect of centrifugation of 2 mL of cHANPs suspension in Corning^®^ Spin-X^®^ UF Concentrators with (a) 30 kDa MWCO at 1000 and 3000 RPM; (b) 50 kDa MWCO at 1000 and 3000 RPM, Figure S5: SEM images of the optimized conditions (a) concentrated phase (b) continuous phase, Table S2: Gd-DTPA quantification in co-loaded cHANPs and ANG-cHANPs and amout of bound angiopep-2 in ANG-cHANPs, Figure S6: T1 distributions of (a) cHANPs; (b) ANG-cHANPs (diluted 1:2) compared to the same concentration of Gd-DTPA free in water, Table S3: T1 measurement of NPs, Figure S7: Stability of cHANPs in culture medium at 37 °C, Figure S8: Comparison of uptake rates by U87 cells of cHANPs and ANG-cHANPs in short times, Figure S9: MTT assay on U87-MG cells incubated with 10 µM of free Irinotecan, Irinotecan in Thera-cHANPs and in Thera-ANG-cHANPs, Figure S10: BCA calibration curve, Figure S11: Calibration curves (a) ATTO 488; (b) ATTO 633, Figure S12: Raw flow cytometry data. Identification of cell population to estimate mean FSC/SSC values of (a) healthy untreated U87; (b) Thera-cHANPs treated U87; (c) Thera-ANG-cHANPs treated U87.

## References

[1] Skalli O., Wilhelmsson U., Orndahl C., Fekete B., Malmgren K., Rydenhag B., Pekny M. (2013). Astrocytoma grade IV (gLioblastoma multiforme) displays 3 subtypes with unique expression profiles of intermediate filament proteins. Hum. Pathol..

[2] Weller M., van den Bent M., Tonn J.C., Stupp R., Preusser M., Cohen-Jonathan-Moyal E., Henriksson R., Le Rhun E., Balana C., Chinot O. (2017). European Association for Neuro-Oncology (EANO) guideline on the diagnosis and treatment of adult astrocytic and oligodendroglial gliomas. Lancet Oncol..

[3] van den Bent M., MacDonald D., Chang S., Vogelbaum M.A., Wen P.Y. (2010). Updated Response Assessment Criteria for High-Grade Gliomas (HGG): Report from the Response Assessment in Neuro-Oncology (RANO) Working Group. Neuro. Oncol..

[4] Lee S.Y. (2016). Temozolomide resistance in glioblastoma multiforme. Genes Dis..

[5] Castro B.A., Aghi M.K. (2014). Bevacizumab for glioblastoma: Current indications, surgical implications, and future directions. Neurosurg. Focus.

[6] Jain R.K., Stylianopoulos T. (2010). Delivering nanomedicine to solid tumors. Nat. Rev. Clin. Oncol..

[7] Boucher Y., Salehi H., Witwer B., Harsh G.R., Jain R.K. (1997). Interstitial fluid pressure in intracranial tumours in patients and in rodents. Br. J. Cancer.

[8] Jain K.K. (2012). Nanobiotechnology-based strategies for crossing the blood-brain barrier. Nanomedicine.

[9] Quail D.F., Joyce J.A. (2017). The Microenvironmental Landscape of Brain Tumors. Cancer Cell.

[10] Ulrich T.A., Pardo E.M.D., Kumar S. (2009). The Mechanical Rigidity of the Extracellular Matrix Regulates the Structure, Motility, and Proliferation of Glioma Cells. Cancer Res..

[11] Seano G., Nia H.T., Emblem K.E., Datta M., Ren J., Krishnan S., Kloepper J., Pinho M.C., Ho W.W., Ghosh M. (2019). Solid stress in brain tumours causes neuronal loss and neurological dysfunction and can be reversed by lithium. Nat. Biomed. Eng..

[12] Schiffer D., Annovazzi L., Casalone C., Corona C., Mellai M. (2019). Glioblastoma: Microenvironment and Niche Concept. Cancers.

[13] Demeule M., Régina A., Jodoin J., Laplante A., Dagenais C., Berthelet F., Moghrabi A., Béliveau R. (2002). Drug transport to the brain: Key roles for the efflux pump P-glycoprotein in the blood-brain barrier. Vasc. Pharmacol..

[14] Seano G. (2018). Targeting the perivascular niche in brain tumors. Curr. Opin. Oncol..

[15] Yang D.W. (2016). Standardized MRI assessment of high-grade glioma response: A review of the essential elements and pitfalls of the RANO criteria. Neuro. Oncol. Pract..

[16] da Cruz L.C.H., Rodriguez I., Domingues R.C., Gasparetto E.L., Sorensen A.G. (2011). Pseudoprogression and Pseudoresponse: Imaging Challenges in the Assessment of Posttreatment Glioma. Am. J. Neuroradiol..

[17] Zikou A., Sioka C., Alexiou G.A., Fotopoulos A., Voulgaris S., Argyropoulou M.I. (2018). Radiation Necrosis, Pseudoprogression, Pseudoresponse, and Tumor Recurrence: Imaging Challenges for the Evaluation of Treated Gliomas. Contrast Media Mol. Imaging.

[18] Prager A.J., Martinez N., Beal K., Omuro A., Zhang Z., Young R.J. (2015). Diffusion and Perfusion MRI to Differentiate Treatment-Related Changes Including Pseudoprogression from Recurrent Tumors in High-Grade Gliomas with Histopathologic Evidence. Am. J. Neuroradiol..

[19] Wake N., Chandarana H., Rusinek H., Fujimoto K., Moy L., Sodickson D.K., Kim S.G. (2018). Accuracy and precision of quantitative DCE-MRI parameters: How should one estimate contrast concentration?. Magn. Reson. Imaging.

[20] Man F., Lammers T., de Rosales R.T.M. (2018). Imaging Nanomedicine-Based Drug Delivery: A Review of Clinical Studies. Mol. Imaging Biol..

[21] Lux F., Tran V.L., Thomas E., Dufort S., Rossetti F., Martini M., Truillet C., Doussineau T., Bort G., Denat F. (2018). AGuIX^®^ from bench to bedside—Transfer of an ultrasmall theranostic gadolinium-based nanoparticle to clinical medicine. Br. J. Radiol..

[22] Lin G., Zhang X.J.A., Kumar S.R., Mark J.E. (2010). Modification of Polysiloxane Networks for Biocompatibility. Mol. Cryst. Liq. Cryst..

[23] Danhier F. (2016). To exploit the tumor microenvironment: Since the EPR effect fails in the clinic, what is the future of nanomedicine?. J. Control. Release.

[24] Stylianopoulos T., Jain R.K. (2015). Design considerations for nanotherapeutics in oncology. Nanomed. Nanotechnol. Biol. Med..

[25] Sarkaria J.N., Hu L.S., Parney I.F., Pafundi D.H., Brinkmann D.H., Laack N.N., Giannini C., Burns T.C., Kizilbash S.H., Laramy J.K. (2018). Is the blood-brain barrier really disrupted in all glioblastomas? A critical assessment of existing clinical data. Neuro-Oncol..

[26] Park J.B., Kwak H.J., Lee S.H. (2008). Role of hyaluronan in glioma invasion. Cell Adhes. Migr..

[27] Kim Y., Kumar S. (2014). CD44-Mediated Adhesion to Hyaluronic Acid Contributes to Mechanosensing and Invasive Motility. Mol. Cancer Res..

[28] Vecchione D., Grimaldi A.M., Forte E., Bevilacqua P., Netti P.A., Torino E. (2017). Hybrid Core-Shell (HyCoS) Nanoparticles produced by Complex Coacervation for Multimodal Applications. Sci. Rep..

[29] Russo M., Bevilacqua P., Netti P.A., Torino E. (2016). A Microfluidic Platform to design crosslinked Hyaluronic Acid Nanoparticles (cHANPs) for enhanced MRI. Sci. Rep..

[30] Russo M., Ponsiglione A.M., Forte E., Netti P.A., Torino E. (2017). Hydrodenticity to enhance relaxivity of gadolinium-DTPA within crosslinked hyaluronic acid nanoparticles. Nanomedicine.

[31] De Sarno F., Ponsiglione A.M., Grimaldi A.M., Netti P.A., Torino E. (2019). Effect of crosslinking agent to design nanostructured hyaluronic acid-based hydrogels with improved relaxometric properties. Carbohydr. Polym..

[32] De Sarno F., Ponsiglione A.M., Russo M., Grimaldi A.M., Forte E., Netti P.A., Torino E. (2019). Water-Mediated Nanostructures for Enhanced MRI: Impact of Water Dynamics on Relaxometric Properties of Gd-DTPA. Theranostics.

[33] Tammaro O., di Polidoro A.C., Romano E., Netti P.A., Torino E. (2020). A Microfluidic Platform to design Multimodal PEG—Crosslinked Hyaluronic Acid Nanoparticles (PEG-cHANPs) for diagnostic applications. Sci. Rep..

[34] Sona M.M., Viswanadh M.K., Singh R.P., Agrawal P., Mehata A.K., Pawde D.M., Narendra, Sonkar R., Muthu M.S. (2018). Nanotheranostics: Emerging Strategies for Early Diagnosis and Therapy of Brain Cancer. Nanotheranostics.

[35] Taghizadehghalehjoughi A., Hacimuftuoglu A., Cetin M., Ugur A.B., Galateanu B., Mezhuev Y., Okkay U., Taspinar N., Taspinar M., Uyanik A. (2018). Effect of metformin/irinotecan-loaded poly-lactic-co-glycolic acid nanoparticles on glioblastoma: In vitro and in vivo studies. Nanomedicine.

[36] Zhao Y.H., Li D.D., Zhao J.J., Song J.N., Zhao Y.L. (2016). The role of the low-density lipoprotein receptor-related protein 1 (LRP-1) in regulating blood-brain barrier integrity. Rev. Neurosci..

[37] Bu G.J., Maksymovitch E.A., Geuze H., Schwartz A.L. (1994). Subcellular-localization and endocytic function of low-density-lipoprotein receptor-related protein in human glioblastoma cells. J. Biol. Chem..

[38] Maletinska L., Blakely E.A., Bjornstad K.A., Deen D.F., Knoff L.J., Forte T.M. (2000). Human glioblastoma cell lines: Levels of low-density lipoprotein receptor and low-density lipoprotein receptor-related protein. Cancer Res..

[39] Demeule M., Régina A., Che C., Poirier J., Nguyen T., Gabathuler R., Castaigne J.P., Béliveau R. (2008). Identification and design of peptides as a new drug delivery system for the brain. J. Pharmacol. Exp. Ther..

[40] Bertrand Y., Currie J.C., Demeule M., Regina A., Che C., Abulrob A., Fatehi D., Sartelet H., Gabathuler R., Castaigne J.P. (2010). Transport characteristics of a novel peptide platform for CNS therapeutics. J. Cell. Mol. Med..

[41] Xin H.L., Sha X.Y., Jiang X.Y., Zhang W., Chen L.C., Fang X.L. (2012). Anti-glioblastoma efficacy and safety of paclitaxel-loading Angiopep-conjugated dual targeting PEG-PCL nanoparticles. Biomaterials.

[42] Xin H.L., Sha X.Y., Jiang X.Y., Chen L.C., Law K., Gu J.J., Chen Y.Z., Wang X., Fang X.L. (2012). The brain targeting mechanism of Angiopep-conjugated poly(ethylene glycol)-co-poly(epsilon-caprolactone) nanoparticles. Biomaterials.

[43] Russo M., Grimaldi A.M., Bevilacqua P., Tammaro O., Netti P.A., Torino E. (2017). PEGylated crosslinked hyaluronic acid nanoparticles designed through a microfluidic platform for nanomedicine. Nanomedicine.

[44] Stetefeld J., McKenna S.A., Trushar R.P. (2016). Dynamic light scattering: A practical guide and applications in biomedical sciences. Biophys. Rev..

[45] Silva M.D., Cocenza D.S., Grillo R., de Melo N.F.S., Tonello P.S., de Oliveira L.C., Cassimiro D.L., Rosa A.H., Fraceto L.F. (2011). Paraquat-loaded alginate/chitosan nanoparticles: Preparation, characterization and soil sorption studies. J. Hazard. Mater..

[46] Li J.Y., Mooney D.J. (2016). Designing hydrogels for controlled drug delivery. Nat. Rev. Mater..

[47] de Sarno F., Ponsiglione A.M., Torino E. Emerging Use of Nanoparticles in Diagnosis Of Atherosclerosis Disease: A Review. Proceedings of the NanoInnovation Conference and Exhibition (Nanoinnovation).

[48] Salvati A., Nelissen I., Haase A., Aberg C., Moya S., Jacobs A., Alnasser F., Bewersdorff T., Deville S., Luch A. (2018). Quantitative measurement of nanoparticle uptake by flow cytometry illustrated by an interlaboratory comparison of the uptake of labelled polystyrene nanoparticles. Nanoimpact.

[49] Pont L., Balvers R.K., Kloezeman J.J., Nowicki M.O., van den Bossche W., Kremer A., Wakimoto H., van den Hoogen B.G., Leenstra S., Dirven C.M.F. (2015). In vitro screening of clinical drugs identifies sensitizers of oncolytic viral therapy in glioblastoma stem-like cells. Gene Ther..

[50] Pont L., Kleijn A., Kloezeman J.J., van den Bossche W., Kaufmann J.K., de Vrij J., Leenstra S., Dirven C.M.F., Lamfers M.L.M. (2015). The HDAC Inhibitors Scriptaid and LBH589 Combined with the Oncolytic Virus Delta24-RGD Exert Enhanced Anti-Tumor Efficacy in Patient-Derived Glioblastoma Cells. PLoS ONE.

[51] Balvers R.K., Kleijn A., Kloezeman J.J., French P.J., Kremer A., van den Bent M.J., Dirven C.M.F., Leenstra S., Lamfers M.L.M. (2013). Serum-free culture success of glial tumors is related to specific molecular profiles and expression of extracellular matrixassociated gene modules. Neuro-Oncol..

[52] Nguyen V.H., Lee B.J. (2017). Protein corona: A new approach for nanomedicine design. Int. J. Nanomed..

[53] Yu Q.H., Zhao L.X., Guo C.C., Yan B., Su G.X. (2020). Regulating Protein Corona Formation and Dynamic Protein Exchange by Controlling Nanoparticle Hydrophobicity. Front. Bioeng. Biotechnol..

[54] Manzanares D., Cena V. (2020). Endocytosis: The Nanoparticle and Submicron Nanocompounds Gateway into the Cell. Pharmaceutics.

[55] Salvati A., Aberg C., dos Santos T., Varela J., Pinto P., Lynch I., Dawson K.A. (2011). Experimental and theoretical comparison of intracellular import of polymeric nanoparticles and small molecules: Toward models of uptake kinetics. Nanomed. Nanotechnol. Biol. Med..

[56] Jochums A., Friehs E., Sambale F., Lavrentieva A., Bahnemann D., Scheper T. (2017). Revelation of Different Nanoparticle-Uptake Behavior in Two Standard Cell Lines NIH/3T3 and A549 by Flow Cytometry and Time-Lapse Imaging. Toxics.

[57] Monica M. (2005). Cell and tissue autofluorescence research and diagnostic applications. Biotechnol. Annu. Rev..

[58] Kummrow A., Frankowski M., Bock N., Werner C., Dziekan T., Neukammer J. (2013). Quantitative assessment of cell viability based on flow cytometry and microscopy. Cytom. Part A.

[59] Valencia P.M., Farokhzad O.C., Karnik R., Langer R. (2012). Microfluidic technologies for accelerating the clinical translation of nanoparticles. Nat. Nanotechnol..

[60] Blanco E., Shen H., Ferrari M. (2015). Principles of nanoparticle design for overcoming biological barriers to drug delivery. Nat. Biotechnol..

[61] Jain R.K., Di Tomaso E., Duda D.G., Loeffler J.S., Sorensen A.G., Batchelor T.T. (2007). Angiogenesis in brain tumours. Nat. Rev. Neurosci..

