# Supplementary material for: Theranostic Design of Angiopep-2 Conjugated Hyaluronic Acid Nanoparticles (Thera-ANG-cHANPs) for Dual Targeting and Boosted Imaging of Glioma Cells
Source: Cancers (Basel). 2021 Jan 28;13(3):503. doi: 10.3390/cancers13030503 (PMC7865309; doi:10.3390/cancers13030503)
Supplement: Supplementary file 1 [file cancers-13-00503-s001.pdf]

Supplementary Materials

Theranostic Design of Angiopep-2 Conjugated Hyaluronic Acid Nanoparticles (Thera-ANG-cHANPs) for Dual Targeting and Boosted Imaging of Glioma Cells

Angela Costagliola di Polidoro, Giorgia Zambito, Joost Haeck, Laura Mezzanotte, Martine Lamfers, Paolo Antonio Netti and Enza Torino

Microfluidic Set Up - Thera – cHANPs

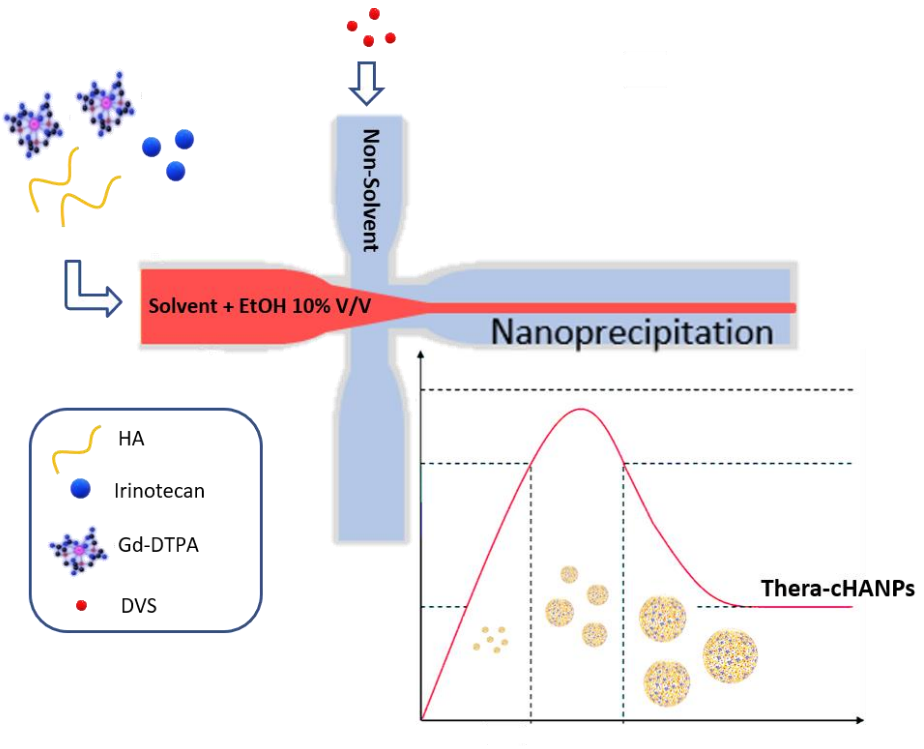

Figure S1 - Schematic representation of Microfluidic experimental set-up for Thera-cHANPs production.

To efficiently encapsulate Irinotecan, a cosolvation strategy is explored in order to overcome limitations due to the low water solubility of Irinotecan (1 mg/mL) (the procedure is reported in the dedicated method section). After production, nanoparticles are gently concentrated via Rotary evaporation in order to reach therapeutic concentrations of Irinotecan.

SEM images of both Thera cHANPs and concentrared Thera-cHANPs are reported to demonstrate the stability of NPs against concentration procedure.

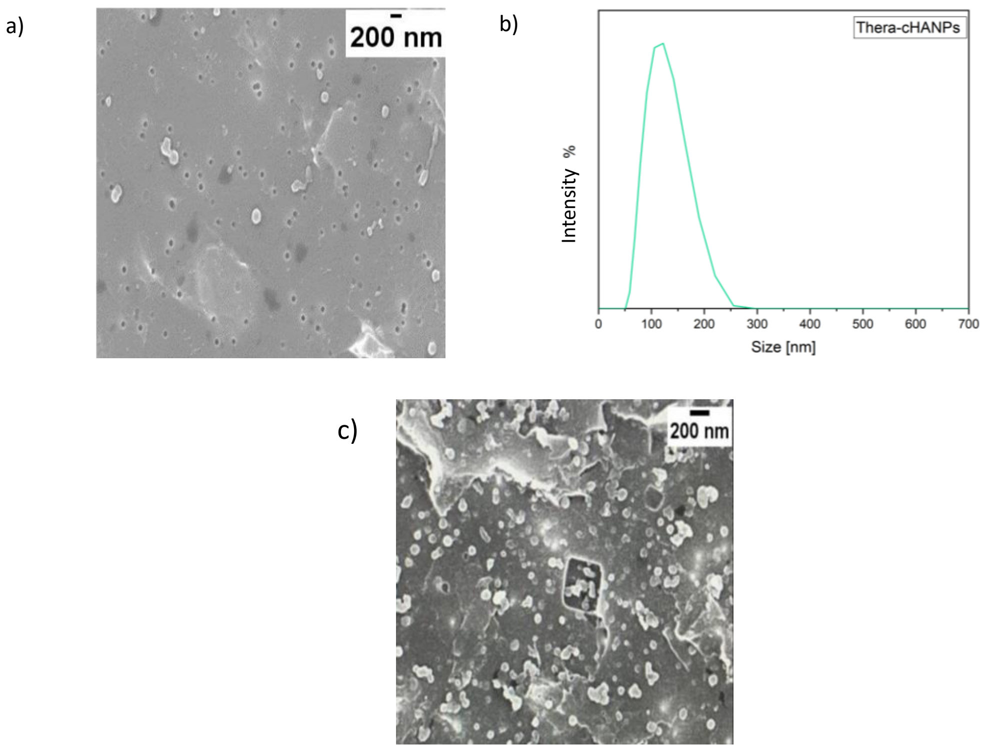

Figure S2 – SEM images of a) Thera-cHANPs; b) PSD of Thera-cHANPs; c) concentrated Thera-cHANPs.

Angiopep-2 bioconjugation reaction optimization

Quantity of peptide

Different concentrations of Angiopep-2 were added to cHANPs suspension and different times of contact were tested in order to assess the best reaction condition.

Table S1 – Different Concentrations of Angiopep-2 and related reaction efficiencies

	**1h**	**2h**	**3h**	**4h**
**[20 ug/mL]**	3.4	2.7	0	8.2
**Efficiency %**	17.2	13.5	0	40.8
**[30 ug/mL]**	3.3	3.5	3.9	10.5
**Efficiency %**	10.9	11.8	12.9	35.1
**[50 ug/mL]**	3.2	4.1	8	27.6
**Efficiency %**	6.4	8.2	15.9	55.2

The amount of peptide bound to nanoparticles exposed at 20 ug/mL of Angiopep-2 after 3h is reported equal to 0, which means that in this measurement, all the absorbances of the triplicate were comparable or slightly lower than the absorbance of bare cHANPs, so that the reported value is equal to zero.

Purification method

Ultracentrifugation (UC)

Via UC, it is not possible to efficiently sediment nanoparticles and remove the excess of peptide without a significant loss of nanoparticles. DLS measurements of sediment and supernatant show similar size distributions (Figure S1), attenuator values and counts per second (kps) revealing similar nanoparticle concentration in both samples, thus inefficient separation.
Figure S2 shows the effect of rpm and centrifugation time in CC of both 30 and 50 kDa MWCO on the volume of the concentrated phase. SEM observations of the concentrated and continuous phase of all the tested conditions allow us to define as optimized purification condition 3000 rpm for 10 min with a 50 kDa filter. In this condition, no aggregation in the concentrated phase and a minimum loss in the continuous phase are observed, Figure S3.

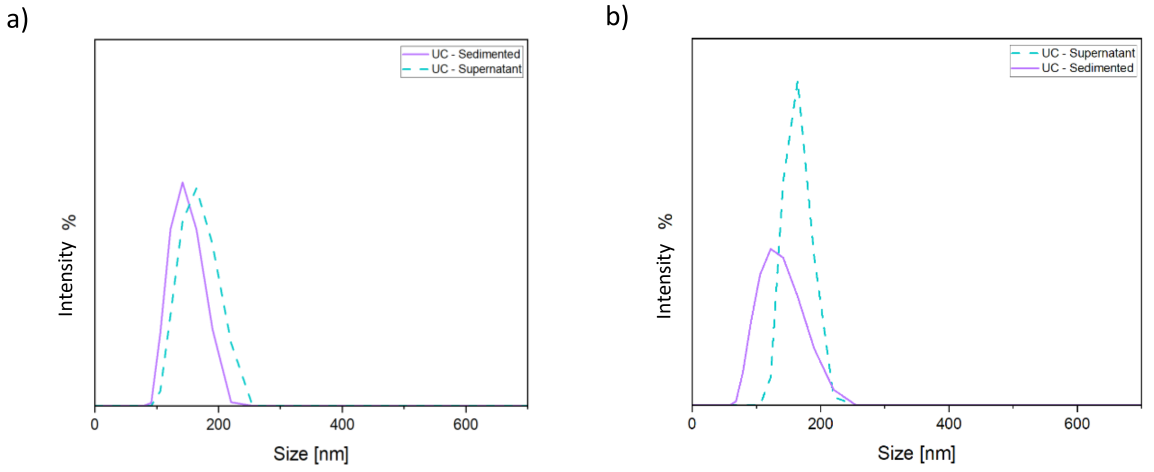

Figure S3 - DLS measurements of sediment and supernatant post ultracentrifugation a) 50000 rpm for 15 min b)70000 rpm for 15 min

Spin X UF concentrators

Study about rpm, time of time of centrifugation, MWCO of the filter.

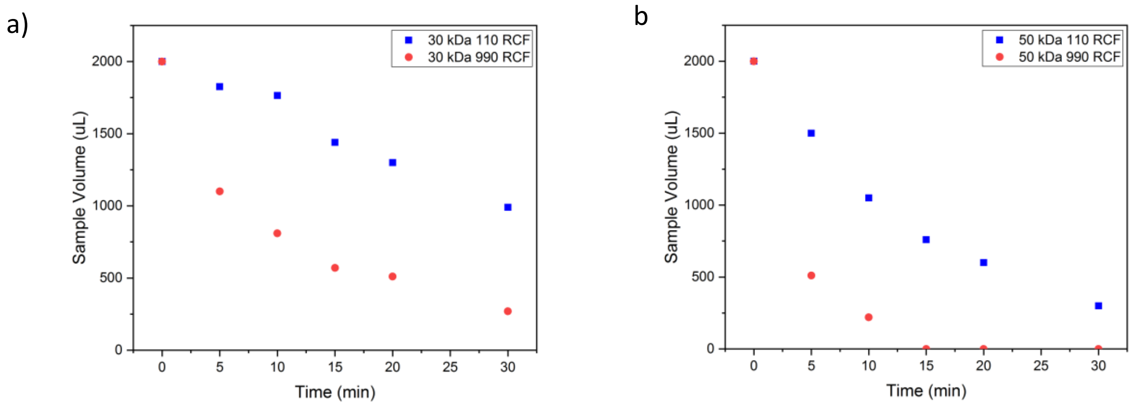

Figure S4 – Effect of centrifugation of 2mL of cHANPs suspension in Corning® Spin-X® UF Concentrators with a) 30 kDa MWCO at 1000 and 3000 rpm; b) 50 kDa MWCO at 1000 and 3000

a) b)

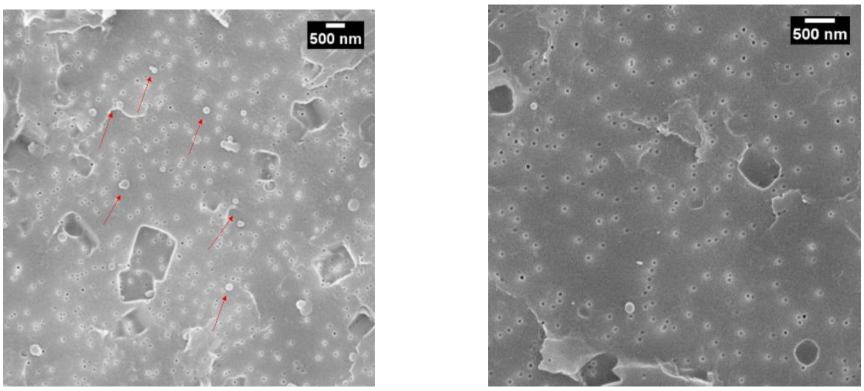

Figure S5 - SEM images of the optimized conditions (a) concentrated phase (b) continuous phase

Characterization of payload agents in Co-loaded ANG-cHANPs

Table S2 – Gd-DTPA quantification in coloaded cHANPs and ANG-cHANPs and amout of bound Angiopep-2 in ANG-cHANPs

	**Gd-DTPA concentration [µM]**	**Gd-DTPA EE%**	**Angiopep-2 Concentration [µg/mL]**
cHANPs	12,02	3,59	--
ANG-cHANPs	5,3	--	27,63
Thera – cHANPs	8,11	2,6	--
Thera – ANG-cHANPs	2,65	--	29,62

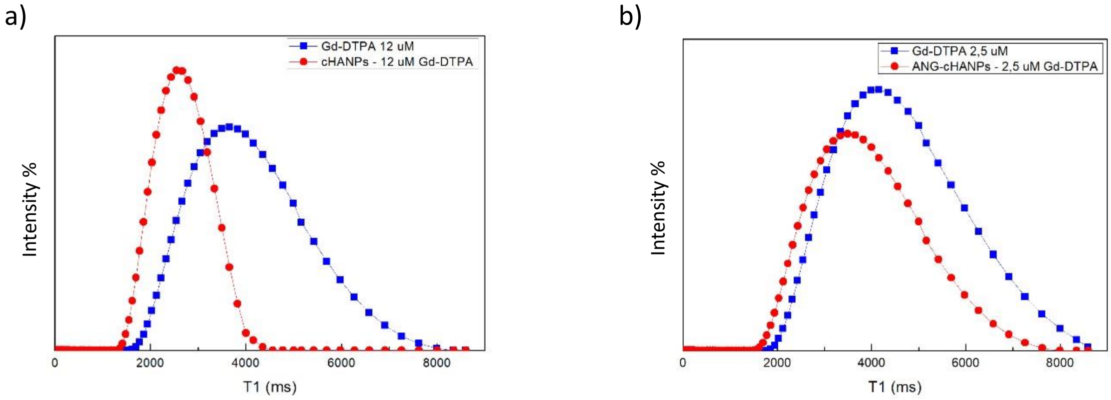

Figure S6 – T1 distributions of (a) cHANPs; (b) ANG-cHANPs (diluted 1:2) compared to the same concentration of Gd-DTPA free in water

Table S3 – T1 measurement of NPs

	T1 [ms]<br>Mean ± st dev.
cHANPs	2385 ± 10
ANG-cHANPs	3003,5 ± 20
Thera-cHANPs	2730 ± 10
Thera-ANG-cHANPs	2790 ± 10

NPs Imaging properties

MRI images of NPs were acquired on a 1T SPECT/MRI system (NanoScan; Mediso Medical Imaging) with a solenoid coil with 35mm diameter in 30 min. Multiple T1 images were acquired using a spin-echo sequence (repetition times 550, 880, 1400, 2000 ms, echo time 80 ms) and T1 maps were calculated using MLE method to fit saturation recovery curves (a*exp(-TR/T1)+c). The other scan parameters included an 80-mm field of view, a 96 × 96 matrix, and 2 mm slice thickness.

T1 weighted images at 37°C by a 1 Tesla clinical MRI scanner are acquired to compare cHANPs at different concentration co-loading Gd-DTPA and Irinotecan. In the following figure a T1 map scale bar is also reported to observe the difference in the relaxation time. Starting from the *top-right* image and moving anticlockwise (from 1 to 4) , it is possible to observe a reduction in the shortening of the T1 signal due to a lower concentration of the Gd-DTPA obtained by the diluition of the cHANPs measured at concentrations from 100 µM to 12,5 µM. Please note that the range of measured concentrations has been chosen to stay in the linear range of relaxivity. These results confirm the ability of the cHANPs to be also used in MRI.

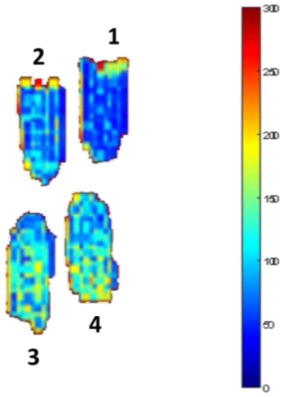

T1 weighted images at 37°C by a 1 Tesla clinical MRI scanner acquired on cHANPS at different Gd-DTPA concentration: (1) ; (2); (3); (4);

Uptake of cHANPs and ANG-cHANPs by U87 cells

cHANPs Stability in culture medium at 37°C up to 24h.

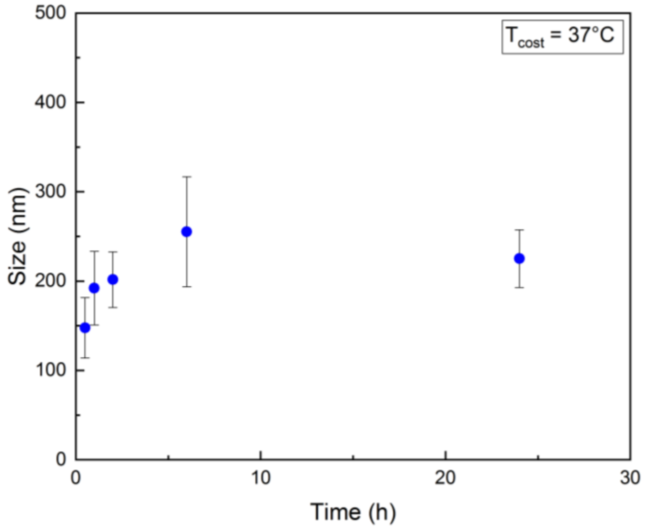

Figure S7 - Stability of cHANPs in culture medium at 37°C

Short times uptake rates of cHANPs and ANG-cHANPs by U87 cells

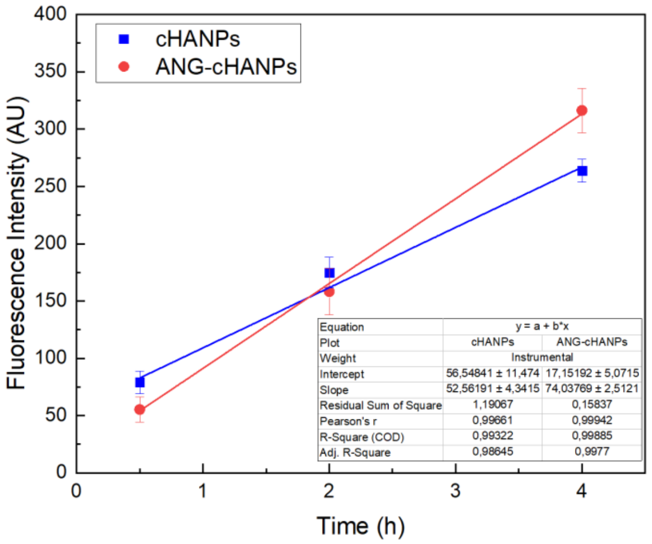

Figure S8 – Comparison of uptake rates by U87 cells of cHANPs and ANG-cHANPs in short times

Cell Viability Assay – MTT

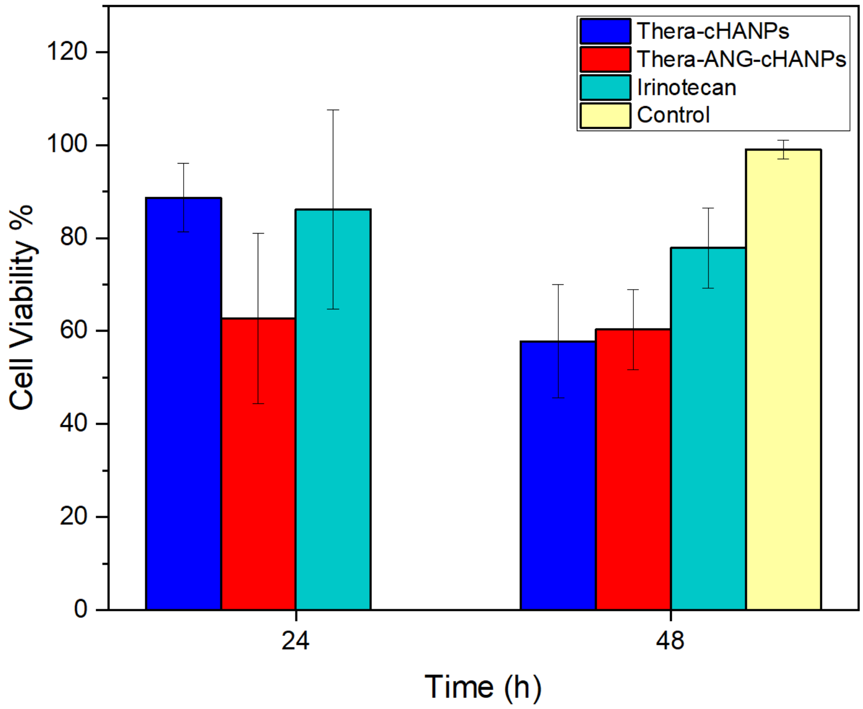

Figure S9 – MTT assay on U87-MG cells incubated with 10 µM of free Irinotecan, Irinotecan in Thera-cHANPs and in Thera-ANG-cHANPs.

Calibration curves

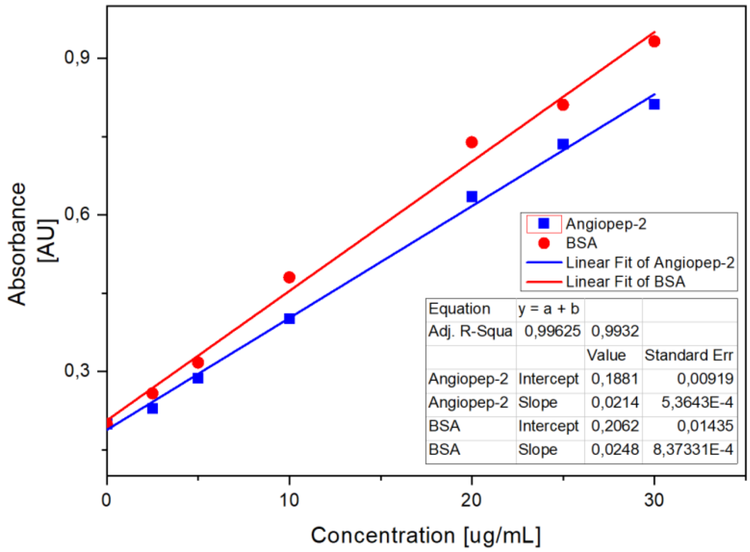

Figure S10 – BCA calibration curve

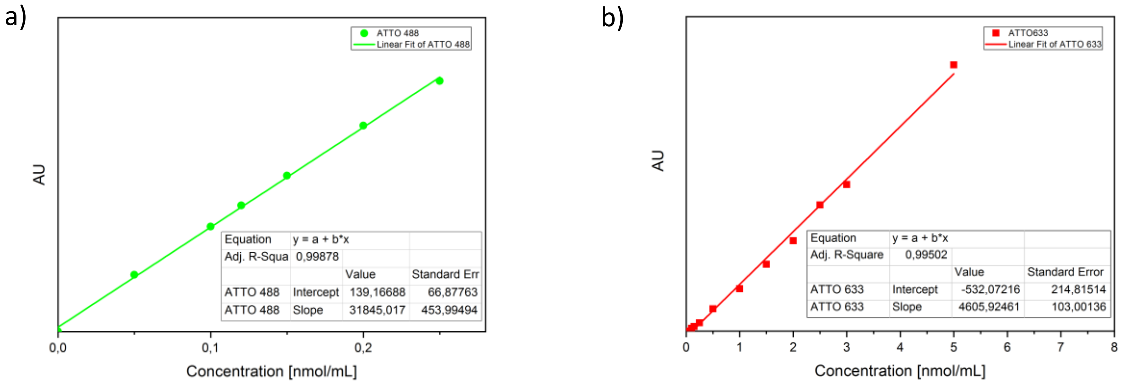

Figure S11 – Calibration curves (a) ATTO 488; (b) ATTO 633

Raw Flow Cytometry data of Thera-cHANPs on U87 cells

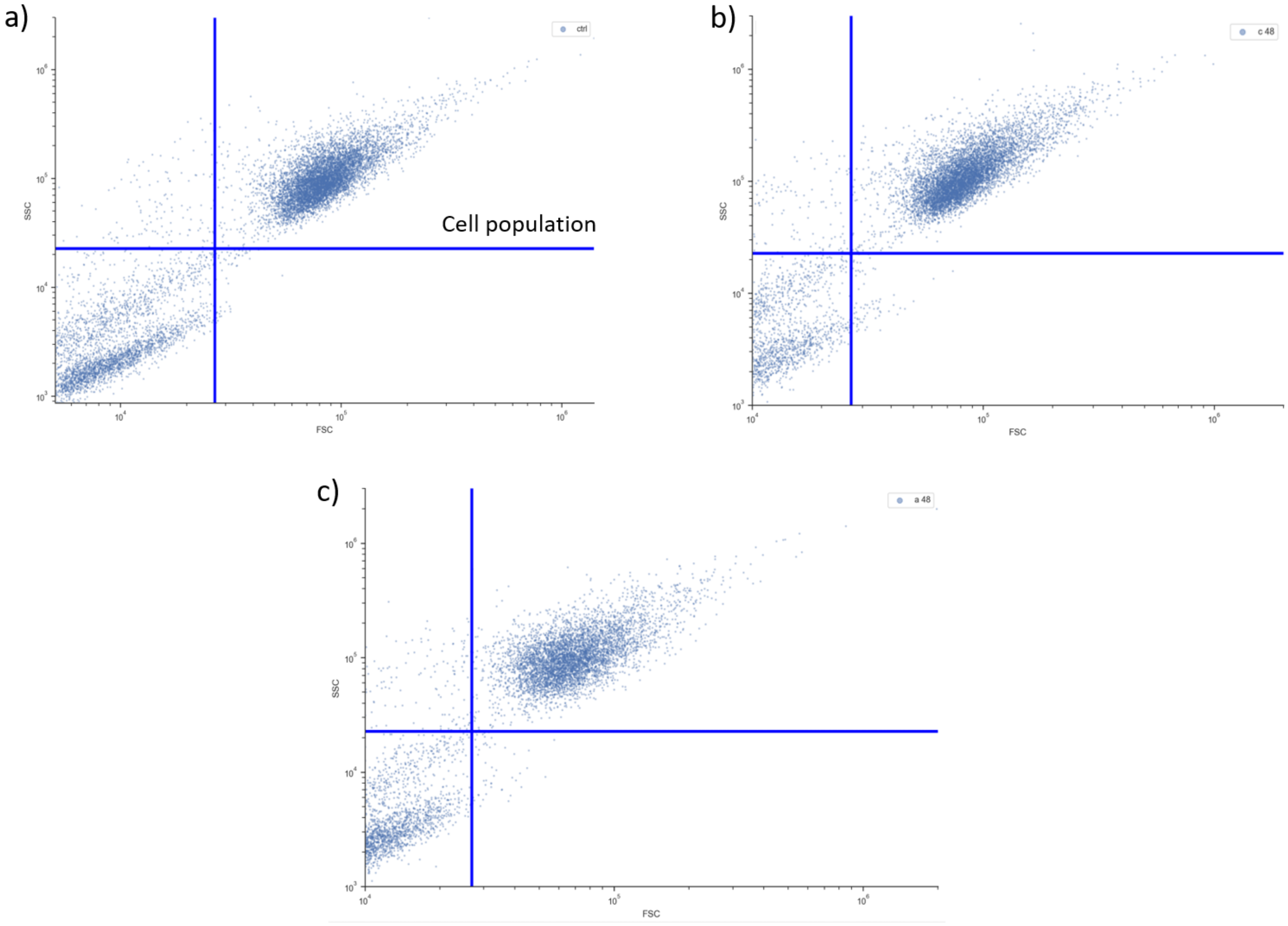

Figure S12 – Raw flow cytometry data. Identification of cell population to estimate mean FSC/SSC values of a) healthy untreated U87; b) Thera-cHANPs treated U87; c) Thera-ANG-cHANPs treated U87.
